# Phylogeography and genomic epidemiology of SARS-CoV-2 in Italy and Europe with newly characterized Italian genomes between February-June 2020

**DOI:** 10.1038/s41598-022-09738-0

**Published:** 2022-04-06

**Authors:** Alessia Lai, Annalisa Bergna, Stefano Toppo, Marina Morganti, Stefano Menzo, Valeria Ghisetti, Bianca Bruzzone, Mauro Codeluppi, Vito Fiore, Emmanuele Venanzi Rullo, Guido Antonelli, Loredana Sarmati, Gaetano Brindicci, Annapaola Callegaro, Caterina Sagnelli, Daniela Francisci, Ilaria Vicenti, Arianna Miola, Giovanni Tonon, Daniela Cirillo, Ilaria Menozzi, Sara Caucci, Francesco Cerutti, Andrea Orsi, Roberta Schiavo, Sergio Babudieri, Giuseppe Nunnari, Claudio M. Mastroianni, Massimo Andreoni, Laura Monno, Davide Guarneri, Nicola Coppola, Andrea Crisanti, Massimo Galli, Gianguglielmo Zehender, SCIRE-SARS-CoV-2 Italian Research Enterprise-Collaborative Group, Claudia Balotta, Carla della Ventura, Marco Schiuma, Enrico Lavezzo, Paolo Fontana, Luca Bianco, Luigi Bertolotti, Laura Manuto, Marco Grazioli, Federico Bianca, Claudia Del Vecchio, Elisa Franchin, Francesco Onelia, Andrea Spitaleri, Francesca Saluzzo, Giovanni Lorenzin, Stefano Pongolini, Erika Scaltriti, Laura Soliani, Patrizia Bagnarelli, Chiara Turchi, Valerio Onofri, Filomena Melchionda, Adriano Tagliabracci, Elisa Burdino, Maria Grazia Milia, Patrizia Caligiuri, Vanessa De Pace, Valentina Ricucci, Alexander Domnich, Simona Boccotti, Leoni Maria Cristina, Giuliana Lo Cascio, Salvatore Rubino, Vincenzo Lai, Giulia Rocca, Rosalba Govoni, Giuseppe Mancuso, Roberta Campagna, Laura Mazzuti, Giuseppe Oliveto, Ombretta Turriziani, Laura Campogiani, Mirko Compagno, Luigi Coppola, Angela Maria Antonia Crea, Giuseppe De Simone, Andrea Di Lorenzo, Ludovica Ferrari, Marco Iannetta, Vincenzo Malagnino, Tiziana Mulas, Benedetta Rossi, Ilaria Spalliera, Simona Tedde, Elisabetta Teti, Pietro Vitale, Marta Zordan, Eugenio Milano, Antonella Lagioia, Rosa Gallitelli, Mario Starace, Carmine Minichini, Alessia Di Fraia, Maddalena Schioppa, Rita Greco, Anna Gidari, Maurizio Zazzi, Filippo Dragoni, Laura Li Puma, Silvia Ronchiadin, Luigi Ruggerone, Dario Russignaga

**Affiliations:** 1 Department of Biomedical and Clinical Sciences Luigi Sacco, University of Milan, Milan, Italy; 2 Pediatric Clinical Research Center Fondazione Romeo ed Enrica Invernizzi, University of Milan, Milan, Italy; 3 Department of Molecular Medicine, University of Padova, Padua, Italy; 4 CRIBI Biotech Center, University of Padova, Padua, Italy; 5 Risk Analyses and Genomic Epidemiology Unit, Istituto Zooprofilattico Sperimentale della Lombardia e dell’Emilia Romagna, Parma, Italy; 6 Department of Biomedical Sciences and Public Health, Virology Unit, Polytechnic University of Marche, Ancona, Italy; 7 Laboratory of Microbiology and Virology, Amedeo di Savoia, ASL Città di Torino, Torino, Italy; 8 Hygiene Unit, IRCCS AOU San Martino-IST, Genoa, Italy; 9 UOC of Infectious Diseases, Department of Oncology and Hematology, Guglielmo da Saliceto Hospital, AUSL Piacenza, Piacenza, Italy; 10 Infectious and Tropical Disease Clinic, Department of Medical, Surgical and Experimental Sciences, University of Sassari, Sassari, Italy; 11 Unit of Infectious Diseases, Department of Experimental and Clinical Medicine, University of Messina, Messina, Italy; 12 Department of Molecular Medicine, University Hospital Policlinico Umberto I, Sapienza University of Rome, Rome, Italy; 13 Infectious Diseases, Tor Vergata University, Rome, Italy; 14 Infectious Diseases Unit, University of Bari, Bari, Italy; 15 Microbiology and Virology Laboratory, ASST Papa Giovanni XXIII, Bergamo, Italy; 16 Department of Mental Health and Public Medicine, University of Campania “Luigi Vanvitelli”, Naples, Italy; 17 Department of Medicine and Surgery, Clinic of Infectious Diseases, “Santa Maria della Misericordia” Hospital, University of Perugia, Perugia, Italy; 18 Department of Medical Biotechnologies, University of Siena, Siena, Italy; 19 Intesa San Paolo Innovation Center-AI LAB, Turin, Italy; 20 Center for Omics Sciences, IRCCS Ospedale San Raffaele, Milan, Italy; 21 Division of Experimental Oncology, IRCCS Ospedale San Raffaele, Milan, Italy; 22 Division of Immunology, Transplantation and Infectious Disease, IRCCS Ospedale San Raffaele, Milan, Italy; 23 Department of Health Sciences (DISSAL), University of Genoa, Genoa, Italy; 24 UOC of Microbiology, Department of Clinical Pathology, Guglielmo da Saliceto Hospital, AUSL Piacenza, Piacenza, Italy; 25 Department of Public Health and Infectious Diseases, University Hospital Policlinico Umberto I, Sapienza University of Rome, Rome, Italy; 26 Microbiology and Virology Diagnostic Unit, Padua University Hospital, Padua, Italy; 27 Department of Life Science, Imperial College London, South Kensington Campus Imperial College Road, London, SW7 AZ UK; 28 CRC-Coordinated Research Center “EpiSoMI”, University of Milan, Milan, Italy; 29 Research and Innovation Centre, Fondazione Edmund Mach, San Michele All’Adige, Trento, Italy; 30 Department of Veterinary Science, University of Studies of Torino, Turin, Italy; 31 Emerging Bacterial Pathogens Unit IRCCS Ospedale San Raffaele, Milan, Italy; 32 Department of Biomedical Science, University of Sassari, Sassari, Italy; 33 Department of Human Pathology in Adulthood and Childhood “Gaetano Barresi” Division of Microbiology, University of Messina, Messina, Italy; 34 AORN S. Anna E S. Sebastiano Caserta, Caserta, Italy; 35 Prevention and Protection Service, INTESA s.p.a., Milan, Italy

**Keywords:** Evolutionary genetics, Molecular evolution, Microbiology, Molecular biology

## Abstract

The aims of this study were to characterize new SARS-CoV-2 genomes sampled all over Italy and to reconstruct the origin and the evolutionary dynamics in Italy and Europe between February and June 2020. The cluster analysis showed only small clusters including < 80 Italian isolates, while most of the Italian strains were intermixed in the whole tree. Pure Italian clusters were observed mainly after the lockdown and distancing measures were adopted. Lineage B and B.1 spread between late January and early February 2020, from China to Veneto and Lombardy, respectively. Lineage B.1.1 (20B) most probably evolved within Italy and spread from central to south Italian regions, and to European countries. The lineage B.1.1.1 (20D) developed most probably in other European countries entering Italy only in the second half of March and remained localized in Piedmont until June 2020. In conclusion, within the limitations of phylogeographical reconstruction, the estimated ancestral scenario suggests an important role of China and Italy in the widespread diffusion of the D614G variant in Europe in the early phase of the pandemic and more dispersed exchanges involving several European countries from the second half of March 2020.

## Introduction

SARS-CoV-2 was first described in Wuhan city, China, likely resulting from adaptation of an animal virus to humans and spread rapidly around the world, causing > 170 million documented infections and > 3.5 million deaths at the end of May 2021 (https://gisanddata.maps.arcgis.com/apps/opsdashboard/index.html#/bda7594740fd40299423467b48e9ecf6). Italy was the first European country to experience a major SARS-CoV-2 disease (COVID-19) epidemic, with a first wave of transmission characterized by a relatively high number of deaths starting from February 20, in Codogno, Lombardy^1^. A few weeks later, the first lockdown and other containment measures, such as quarantine of travellers returning from high-risk areas, reduced the number of COVID-19 cases in Italy and prevented the escalation of clusters of community transmission.

The COVID-19 pandemic represents an unprecedented challenge for global public health with the continuous emergence of new genetic variants of the virus^2^ and the related implications such as their potentially increased pathogenicity or transmissibility and, possibly, vaccine escape. Notwithstanding a unique proofreading activity among RNA viruses^3^, SARS-CoV-2 has been exploring its genetic space due to an exceedingly large number of transmissions and replicative cycles, with an estimated evolutionary rate around 2 mutations per month. Indeed, notable genomic variability can be observed among all viral sequences submitted to the GISAID database, which have been grouped into two main lineages, A and B, each containing a growing number of sub-lineages^4^. Both lineages likely separated early during the Wuhan outbreak, with lineage B now being more widely distributed. In this context, the establishment of surveillance networks at national and international level is necessary to trace the pandemic and inform the appropriate public health interventions.

Presently no comprehensive data are available to establish the lineage of SARS-CoV-2 strains circulating in Italy and their population dynamics, although regional data have been published for Sardinia^5^, Lombardy^6^ and Abruzzo^7^. The short time since its identification and the limited number of sequences available in public databases makes it difficult to understand the biological significance of the mutations observed so far, whether they are the product of adaptive selection^8^ or rather the result of genetic drift due to the high level of genetic variation (http://virological.org/t/response-to-on-the-origin-and-continuing-evolution-of-sars-cov-2/418). In addition, as Italy can be considered the first and one of the main incubators for the spread of the epidemic in Europe and in the United States, the analysis of SARS-CoV-2 molecular epidemiology since the first phases of the epidemic in this country is of particular interest for unravelling the first evolutionary steps of the virus outside China and its adaptation to western countries. In this context, the reconstruction of the spatial and temporal dynamics is fundamental to understand the origin and evolution of SARS-CoV-2 from the ancestral strains to the new variants.

In this study, viral sequences have been analysed for mutations and phylogeny, in comparison to national and international SARS-CoV-2 genomes, to hypothesize the route of arrival to Italy, the subsequent dispersion and further spread to other countries. Major SARS-CoV-2 infection clusters in Italy were identified and characterized and their role in the international virus spread was assessed by using phylogenetic analyses. In addition, the spatiotemporal SARS-CoV-2 dynamics in Italy was investigated by a relatively new maximum likelihood approach for ancestral character reconstruction, by combining the reconstruction relative to the sampling location with the evolutionary lineage.

## Results

A total of 192 SARS-CoV-2-Italian genomes were newly generated for this study. Travel history was available for 137 (71.3%) patients. All of them reported no international travel in the two weeks preceding the onset of symptoms. One case of contact with a traveller from Bangladesh was reported. Main patients’ information is reported in Table 1.

**Table 1:** Characteristics of the studied populations.

		Sequences (n = 192)
Age	Median (min–max)	68 (9–99)
Gender	M	89
	F	76
Region	Apulia	9
	Campania	4
	Emilia Romagna	14
	Lazio	20
	Liguria	15
	Lombardy	10
	Marche	11
	Piedmont	17
	Sardinia	13
	Sicily	12
	Umbria	2
	Veneto	65
Travel History	Yes	0
	No	121
	n.a.*	16

*n.a.: not available.

### Analysis of the Italian dataset

#### Genomic diversity on the basis of the lineage/clade classification

The most prevalent lineages were B.1 (n = 222, 47.7%, including 32 lineages derived from B.1 such as B.1.76, B.1.91, B.1.104, B.1.142, B.1.153, B.1.177, B.1.179, B.1.222, B.1.225, B.1.356, B.1.610) and B.1.1 (n = 141, 30.3%, including 19 lineages derived from B.1.1 such as B.1.1.28, B.1.1.61, B.1.1.161, B.1.1.202, B.1.1.232, B.1.1.331 and B.1.1.372) followed by the lineages B (n = 73, 15.7%) and B.1.1.1 (n = 29, 6.2%). The Nextclade classification showed a high prevalence of the clades 20A (n = 207, 44.5%) and 20B (n = 141, 30.3%), followed by 19A (n = 84, 18.1%), and 20D (n = 29, 6.2%). Only 4 strains were clade 20C (0.9%).

**Figure 1:**
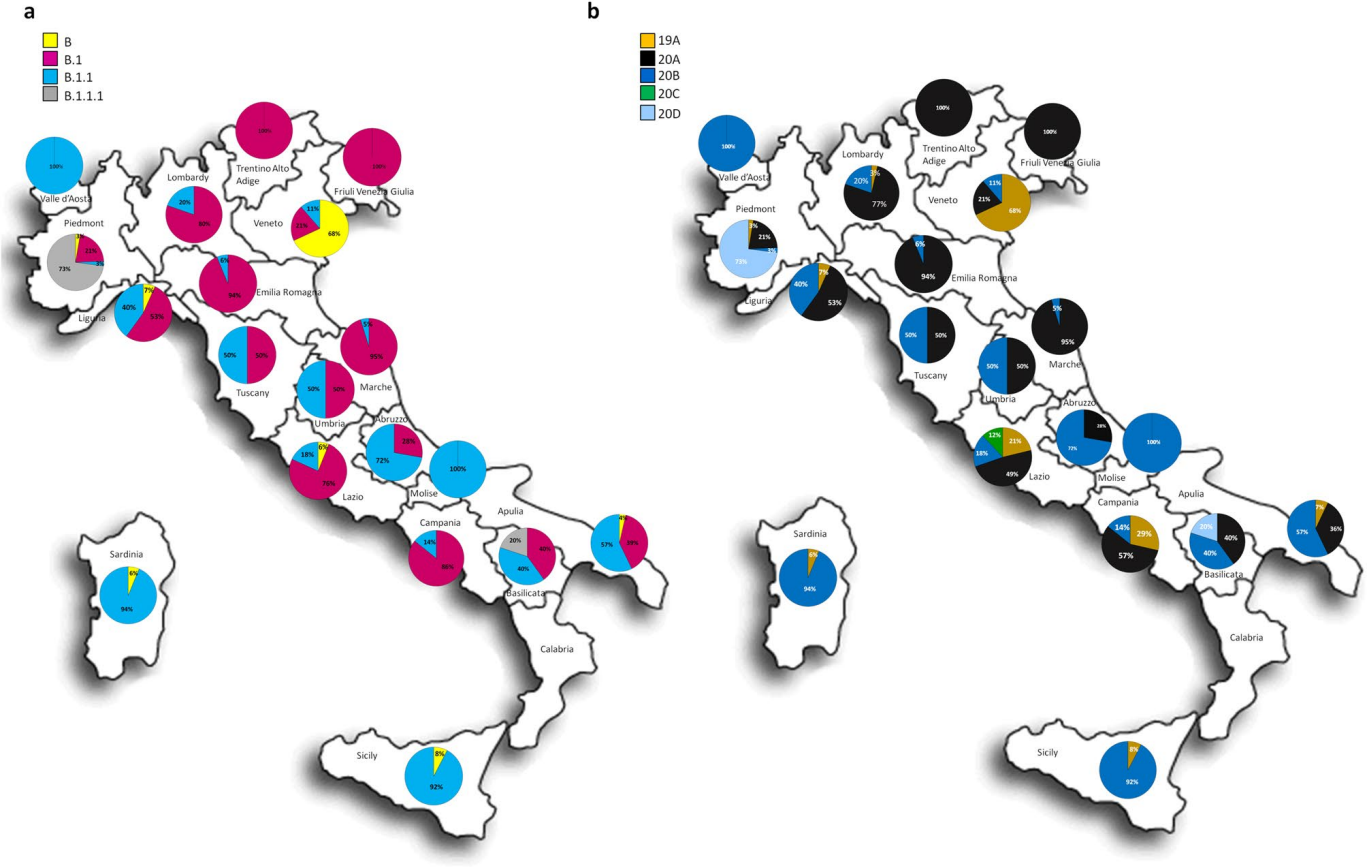
Spatial distribution of lineages and clades. (**a**, **b**) Map of Italy reporting the lineage distribution (**a**) and the clade assignment (**b**) in every region.

The geographical distribution of the SARS-CoV-2 lineages/clades in Italy (Fig. 1) showed several different epidemiological patterns. Some regions mainly in Northern-Central Italy (Friuli Venezia Giulia, Marche, Emilia Romagna, Lombardy, Lazio) showed a high prevalence of B.1/20A (between 70 and 100%). Other regions, mainly in the Central-Southern Italy (Sardinia, Sicily, Abruzzo, Apulia) had the highest prevalence of B.1.1/20B (from 57% to more than 90%). Other regions showed an equal proportion of both lineages (Basilicata, Liguria, Tuscany, Umbria). Two regions had a unique pattern: Veneto, in which the most prevalent lineage was B/19A (66/97, 68%) and Piedmont, showing 73% (27/37) of B.1.1.1/20D lineage.

A change in the prevalence of the SARS-CoV-2 lineages between February and May was observed. The most frequently detected lineages were B/19A and B.1/20A in February and first half of March, representing 88% of all the genomes obtained in that period of time. Subsequently, starting from the second half of March, B.1.1/20B and other lineages (B.1.1.1/20D) became more prevalent (60.7% between 15 and 31 March, 46.2% in April, 51.6% in May).

#### Genetic distances and mutation analyses

The overall mean p-distance between all the Italian isolates was 3.9 (SE: 0.4) s/10,000 nts corresponding to a mean of 10.1 (SE: 1.01) substitutions per genome. Genetic distance remained small with a mean of 10.23 (SE: 1.09) substitutions, of which 3.13 (SE:0.59) were synonymous and 6.85 (SE:0.79) non-synonymous. A higher heterogeneity was observed in sequences from Piedmont (20.4, SE: 1.6) and Sicily (18.4, SE: 1.2) compared to other regions. Interestingly, an increasing number of differences over time was recorded, from 5.7 (SE: 0.81) in February to 20.1 (SE: 1.1) in May.

Seventeen amino acid substitutions were present in more than 10% of the Italian isolates but only one of them was in the spike protein (D614G). No mutations were observed in the receptor binding domain (RBD) in the whole Italian sequence dataset. Only eleven B lineage sequences in the whole dataset, all from Veneto (clade 19A), carried T1543I in orf1a. Overall, the B sequences showed a distinct mutations pattern from those of other lineages, including mutations L3606F, G251V in orf1a and orf3a, respectively. The B.1.1.1 lineage presented additional substitutions in comparison with B.1 and B.1.1 lineages such as T1246I in orf1a in all isolates. Table 2 shows the most frequent amino acid substitutions stratified by lineage and clade.

**Table 2:** Aminoacid substitutions found in more than 10% of sequences stratified according to lineage and clade.

Gene		B n = 73 (%)	B.1 n = 222 (%)	B.1.1 n = 141 (%)	B.1.1.1 n = 29 (%)	19A n = 85 (%)	20A n = 207 (%)	20B n = 141 (%)	20C n = 4 (%)	20D n = 29 (%)
ORF1a^a^	T265I	–	–	–	–		–	–	4 (100)	–
	T1246I	–	–	–	29 (100)		–	–	–	29 (100)
	T1543I	11 (15.6)	–	–	–	11 (13.1)	–	–	–	–
	G3278S	–	–	–	–		–	–	–	29 (100)
	M3752L	–	–	–	5 (17.2)		–	–	–	5 (17.2)
	M3752T	–	–	–	5 (17.2)		–	–	–	5 (17.2)
	L3606F	67 (91.8)	–	–	–	69 (82.1)	–	–	–	–
	F3753I	–	–	–	8 (27,6)		–	–	–	8 (27.6)
ORF1b^b^	P314L	–	214 (96.4)	130 (92.2)	29 (100)		207 (100)	130 (92.2)	4 (100)	29 (100)
S^c^	D614G	–	205 (92.3)	18 (84.3)	29 (100)	9 (10.7)	197 (95.2)	118 (83.7)	1 (25)	29 (100)
ORF3a^d^	Q57H	–	–	–	–		–	–	4 (100)	–
	A99V	–	–	–	–		–	–	3 (75)	–
	G251V	67 (91.8)	–	–	–	69 (82.1)	–	–	–	–
M^e^	D3G	–	51 (22.9)	–	–	–	49 (23.7)	–	–	
N^f^	R203K	–	–	140 (99.3)	29 (100)	–	–	140 (99.3)	–	29 (100)
	G204R	–	–	140 (99.3)	29 (100)	–	–	140 (99.3)	–	29 (100)
ORF14^g^	G50R	–	–	138 (98.6)	29 (100)	–	–	138 (97.9)	–	29 (100)

^a^Open Reading Frames 1a.

^b^Open Reading Frames 1b.

^c^Spike gene.

^d^Open Reading Frames 3a.

^e^Membrane gene.

^f^Nucleocapsid gene.

^g^Open Reading Frames 14.

#### Phylogenetic analysis by ML and Bayesian methods

**Figure 2:**
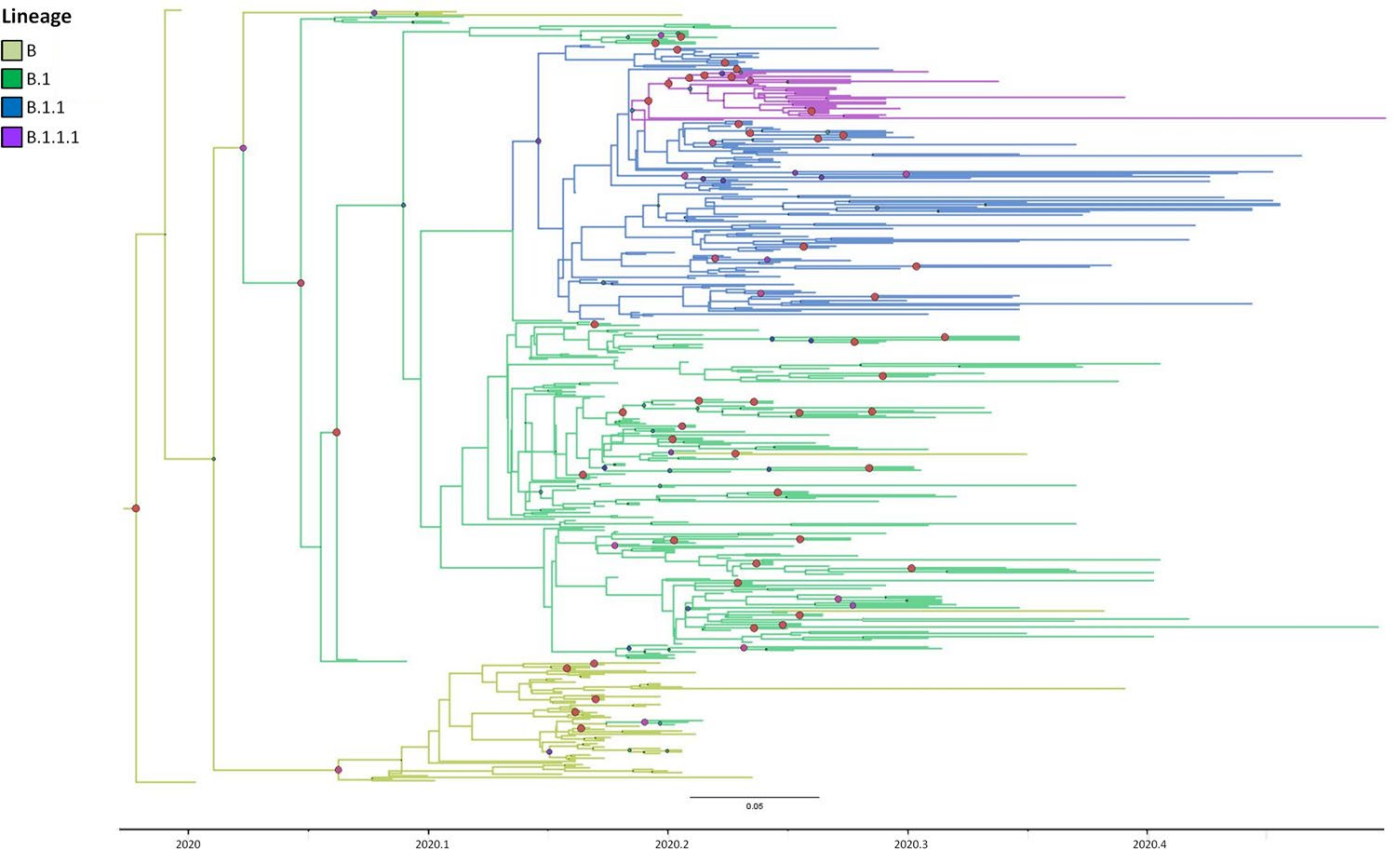
SARS-CoV-2 Bayesian phylogeographic tree of 479 strains. Large red and purple circles indicate highest posterior probability ranging from 1 to 0.9. The branches are coloured based on the most probable lineage of the descendent nodes.

The phylogenetic analysis by Bayesian method assigning each tip to its lineage showed 4 large highly significant clades corresponding to the main circulating lineages in Italy (B, B.1, B.1.1, and B.1.1.1) (Fig. 2). B1, B.1.1 and B.1.1.1 were nested into each other, while B segregated independently. Chinese sequences tended to segregate at the outgroup of the Italian clades within B and B.1 lineages. The estimation of the tMRCAs of the main clades suggested that B lineage spread to Italy in the last week of January 2020, lineage B.1.1 emerged later, in mid-February and B.1.1.1 was the latest, spreading in early March. ML analysis showed similar tMRCAs but with broader confidential intervals (Table 3).

**Table 3:** Time of the Most Recent Common Ancestor (tMRCA) estimates and confidence intervals (CI) of the mains lineages.

Node	Maximum likelihood			Bayesian			
	Median	CI_low	CI_up	Median	CI_low	CI_up	spp*
Tree root	17/12/2019	05/11/2019	28/12/2019	20/12/2019	09/12/2019	28/12/2019	1
B	24/12/2019	30/11/2019	10/01/2020	04/01/2020	28/12/2020	04/01/2020	0.82
B IT	29/01/2020	20/01/2020	29/01/2020	19/01/2020	08/01/2020	26/01/2020	0.92
B.1	24/12/2019	30/11/2019	10/01/2020	15/01/2020	09/01/2020	23/01/2020	0.95
B.1 IT**	24/01/2020	13/01/2020	24/01/2020	19/01/2020	16/01/2020	23/01/2020	0.99
B.1.1	12/02/2020	31/01/2020	16/02/2020	17/02/2020	10/02/2020	21/02/2020	0.73
B.1.1.1	22/02/2020	10/02/2020	05/03/2020	03/03/2020	03/03/2020	10/03/2020	0.99

*spp, state posterior probability.

**IT, Italy.

#### Phylogeography in Italy

**Figure 3:**
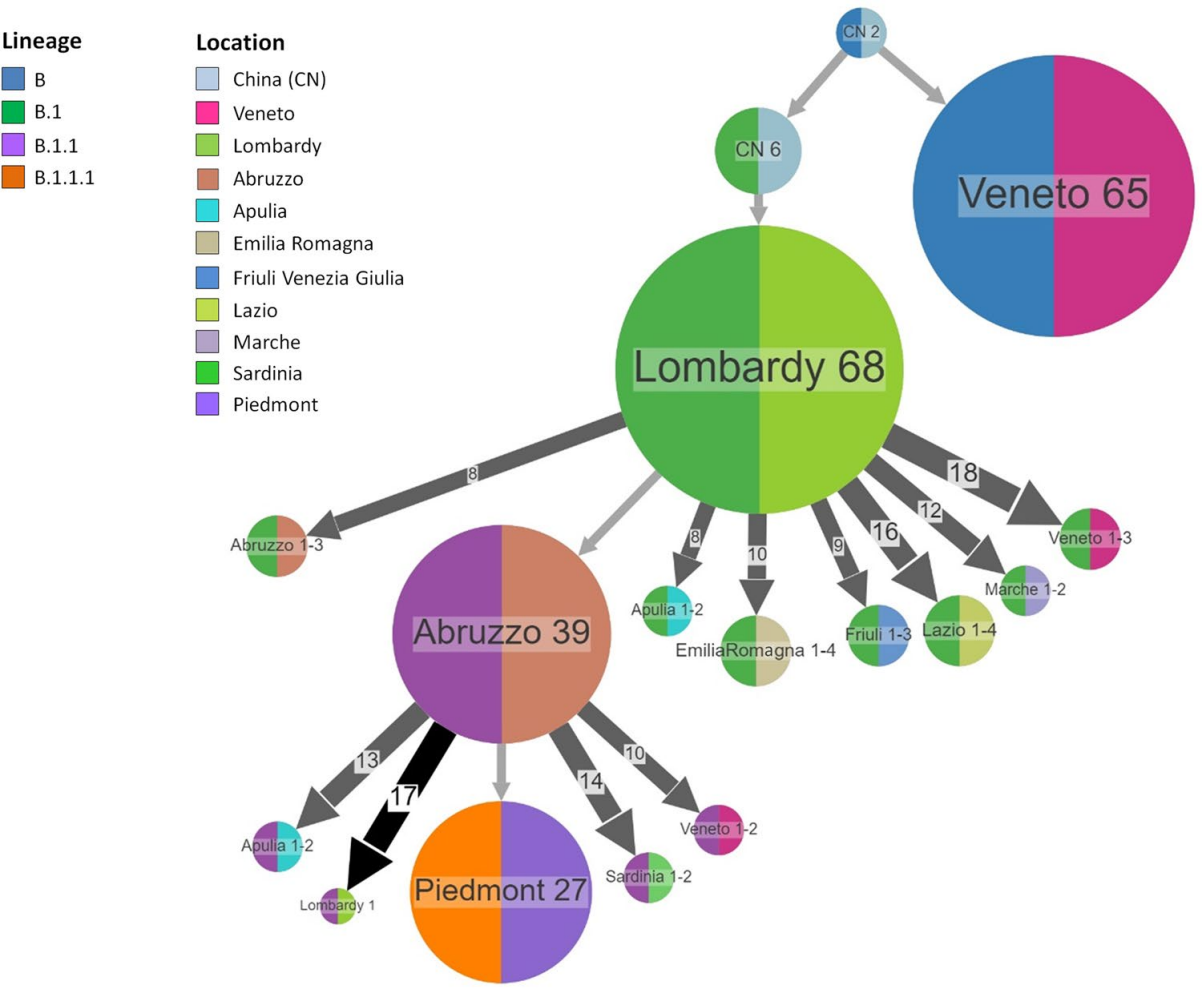
Ancestral reconstruction of SARS-CoV-2 lineages B.1 using the Italian dataset. The figure shows the compressed visualization produced by PastML using marginal posterior probability approximation (MPPA) with an F81-like model. Different colours correspond to different Italian geographical regions and lineages. Numbers inside (or next to) the circles indicate the number of strains assigned to the specific node.

The phylogeography of SARS-CoV-2 identified China as the location of the tree root (Fig. 3 and Supplementary Fig. 1). Four main large clusters were identified. The earliest clusters were in Lombardy and Veneto, directly linked to China, while later (around the second half of March) other clusters appeared in Abruzzo and Piedmont. Combining the phylogeography with the SARS-CoV-2 lineages, the reconstruction of the ancestral state showed that lineage B and B.1 spread from China to Veneto and Lombardy, respectively. While lineage B apparently remained confined to Veneto (and it was successfully extinguished), lineage B.1 further spread from Lombardy to other Italian regions (Veneto, Emilia Romagna, Abruzzo, Marche, Apulia, Friuli Venezia Giulia and Lazio). Lineage B.1.1 spread from central Italy (Abruzzo) to other Italian regions (Veneto, Lombardy, Apulia, Sardinia). Finally, the lineage B.1.1.1 emerged later and remained apparently localized in Piedmont without further spread to other regions.

### Analysis of the international data set

#### Italian clusters

The phylogenetic analysis by ML of the entire dataset including Italian, European and Chinese genomes showed that the majority of the Italian isolates were dispersed in the entire tree. A total of 80 (out of 465, 17.2%) Italian isolates were included in 22 highly supported clusters (Table 4). Of these, 12 (54.5%) were within the lineage B.1, five (22.7%) were B.1.1/20B, three (13.6%) were B.1.1.1/20D and two (9.1%) were B/19A. All but one B.1 clusters were classified as 20A clade. Cluster #19 was the only exception and included four Italian strains classified as clade 20C (all from Rome), showing a mean tMRCA falling in March 2020. Three clusters (13.6%) were singletons (including only single Italian isolates not linked to other Italian sequences), probably corresponding to sporadic introductions followed by limited circulation, while the remaining 19 clusters encompassed at least two Italian isolates, suggesting a local transmission. Thirteen of these (68.4%) included only Italian strains (suggesting a mainly local circulation of this lineage), while 6 (31.6%) included isolates from other European countries, and one of them (B.1) included also one Chinese genome.

**Table 4:** Main characteristics of the identified clusters.

Cluster_ID	Num Seqs	IT^a^	EU^b^	CN^c^	Lineage	Clade	ML^d^ median	CI^e^_low	CI_up	Type of cluster*
1	4	4	0	0	B.1	20A	20/01/2020	08/01/2020	24/01/2020	IT
6	11	2	8	1	B.1	20A	24/01/2020	10/01/2020	21/02/2020	M
22	3	3	0	0	B.1	20A	31/01/2020	11/01/2020	03/03/2020	IT
2	6	6	0	0	B.1	20A	01/02/2020	17/01/2020	10/02/2020	IT
8	3	3	0	0	B.1.1	20B	10/02/2020	28/01/2020	12/03/2020	IT
3	7	5	2	0	B.1	20A	13/02/2020	26/01/2020	22/02/2020	M
7	3	2	1	0	B.1	20A	17/02/2020	17/01/2020	01/03/2020	M
11	3	3	0	0	B.1	20A	20/02/2020	18/01/2020	11/03/2020	IT
10	4	2	2	0	B.1.1	20B	20/02/2020	31/01/2020	12/03/2020	M
9	5	3	2	0	B.1	20A	20/02/2020	26/01/2020	13/03/2020	M
12	6	1	5	0	B.1.1	20B	22/02/2020	06/02/2020	27/02/2020	S
13	3	3	0	0	B.1	20A	23/02/2020	22/01/2020	24/03/2020	IT
5	11	11	0	0	B	19A	24/02/2020	14/02/2020	24/02/2020	IT
4	3	1	0	0	B	19A	24/02/2020	28/01/2020	28/02/2020	S
14	3	3	0	0	B.1	20A	01/03/2020	29/01/2020	01/03/2020	IT
15	11	3	8	0	B.1.1.1	20D	02/03/2020	22/02/2020	02/03/2020	M
17	5	1	4	0	B.1.1.1	20D	02/03/2020	22/02/2020	02/03/2020	S
16	8	8	0	0	B.1.1.1	20D	02/03/2020	22/02/2020	02/03/2020	IT
19	4	4	0	0	B.1	20C	08/03/2020	07/02/2020	11/03/2020	IT
18	3	3	0	0	B.1.1	20B	08/03/2020	06/02/2020	17/03/2020	IT
20	5	5	0	0	B.1.1	20B	18/03/2020	24/02/2020	24/03/2020	IT
21	4	4	0	0	B.1	20A	31/03/2020	05/03/2020	15/04/2020	IT

^a^Italian strains.

^b^European strains, with the exception of Italy.

^c^Chinese strains.

^d^Maximum likelihood.

^e^Confidence Interval.

*Type of cluster: M, mixed; IT, Italian; S, single Italian isolate.

The estimate of the clusters tMRCA by ML method confirmed that the first transmission events in Italy dated around the second half of January and early February. Eighteen clusters had a common ancestor dating before the introduction of the containment measures in our country. In particular, B.1/20A clusters predominated (10/14) at earlier time points (before March) while in March other clades (20B, 20C and 20D) prevailed (6/8). Moreover, the mixed and singleton clusters were prevalent at the beginning, while pure Italian clusters were the only clusters observed after the lockdown. The earliest cluster (#1), was lineage B.1/20A, dated back to average 20/01/2020 (CI95% 08/01–24/01/2020) and included only four North Italian strains: one from Lodi, two from Milan (the locations where autochthonous COVID-19 cases were firstly identified in Italy) and one from Piacenza. The first B.1.1 cluster dated back to 10/02/2020 (CI95% 28/01/2020–12/03/2020) and included 3 Italian isolates from Abruzzo. Three B.1.1.1/20D clusters dated back to 02 March (CI95% 22/02/2020–02/03/2020). Only two small Italian clusters supported by significant bootstraps were observed within the ML tree including B/19A isolates. In particular a single pure Italian cluster included 11 genomes from Veneto (province of Padua), characterized by the substitution T1543I in orf1a, not detected in any of the other B/19A genomes in our international dataset.

#### Phylogeographical analysis in Europe

**Figure 4:**
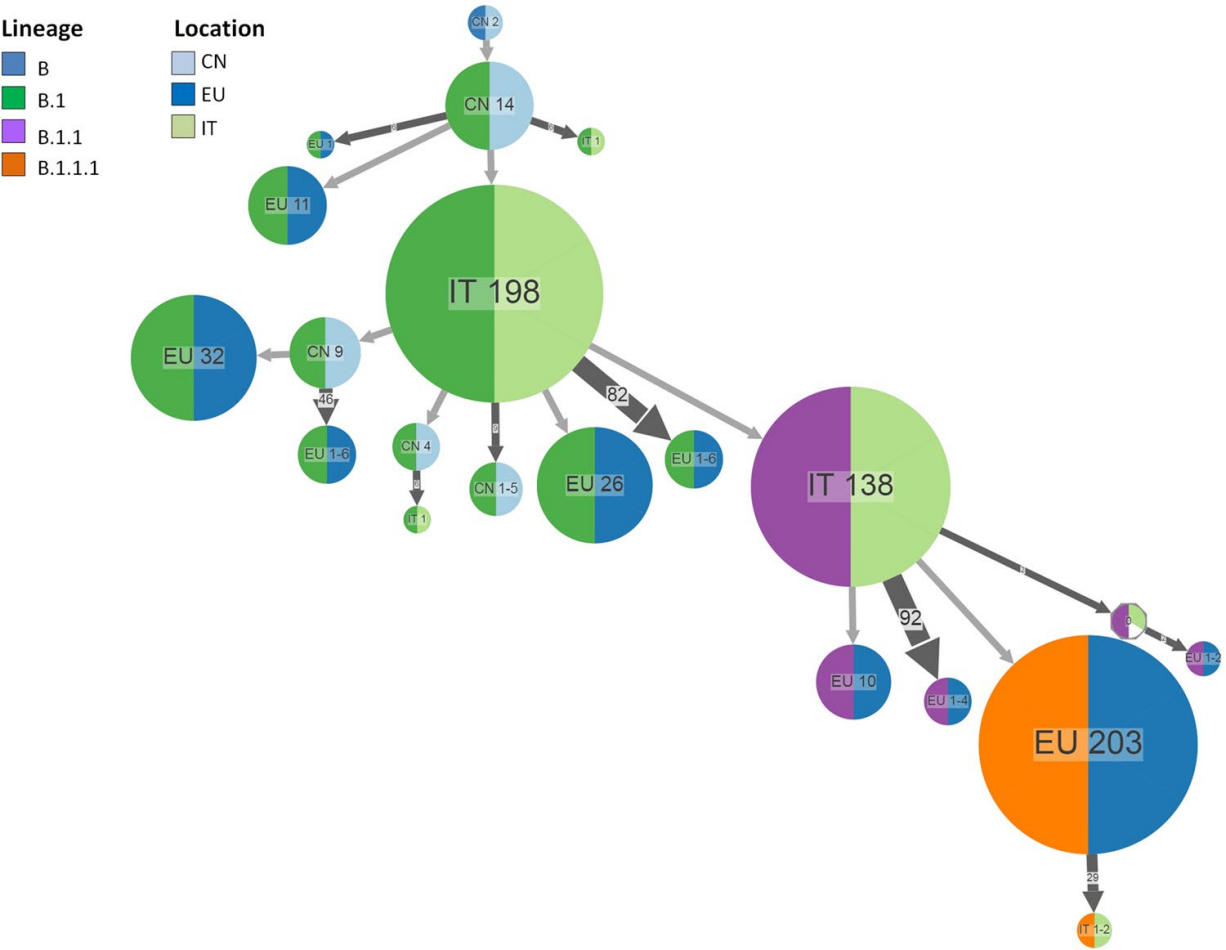
Ancestral reconstruction of SARS-CoV-2 lineages B.1 using the European dataset. The figure shows the compressed visualization produced by PastML using marginal posterior probability approximation (MPPA) with an F81-like model. Different colours correspond to different European countries and lineages. Numbers inside (or next to) the circles indicate the number of strains assigned to the specific node. The joint ancestral scenario (Joint) and maximum a posteriori (MAP) predictions are shown for the uncertain nodes (shown as octagonal icons). CN, China; IT, Italy, EU, Europe.

Combining the ancestral state reconstruction for the location with the lineage (Fig. 4 and Supplementary Fig. 2), the analyses showed that B.1 probably originated in China and spread to several European countries reaching Italy several times, forming a large cluster which included initially 59 (around the first week of March) and finally 198 genomes, and 6 further independent introductions mainly corresponding to a group of genomes characterized only by the substitution D614G but lacking other substitutions, in particular the P314L in the RdRp identifying the clade 20A (lineage B.1, clade 19A).

Starting from Italy, B1/20A spread to other European countries being also reintroduced later to China. A second large Italian cluster, including 138 genomes of lineage B.1.1, emerged from the Italian B.1 cluster. Multiple introductions of B.1.1 were observed from Italy to other European countries. A large cluster (n = 203 genomes) corresponding to B.1.1.1 lineage appeared in Europe in the early March and reached Italy only later (second half of March) (Fig. 4). A total of 7 nodes remained undetermined. A separate analysis conducted distinguishing European countries (rather than considering a single generalized group), generally confirmed this scenario and allowed to reconstruct in greater detail the dispersion of the epidemic in the European countries (Supplementary Fig. 3).

**Figure 5:**
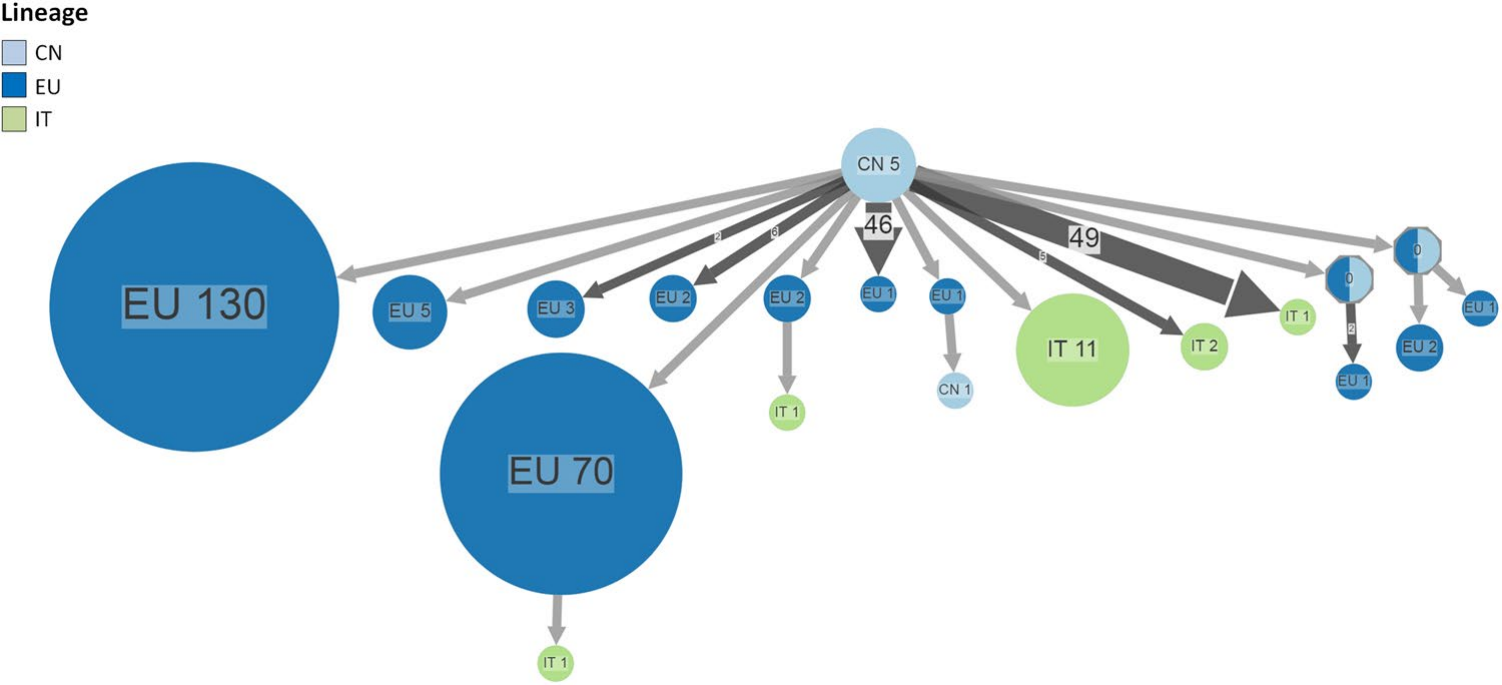
Ancestral reconstruction of SARS-CoV-2 lineages B using the European dataset. The figure shows the compressed visualization produced by PastML using marginal posterior probability approximation (MPPA) with an F81-like model. Different colours correspond to different European countries and lineages. Numbers inside (or next to) the circles indicate the number of strains assigned to the specific node. The joint ancestral scenario (Joint) and maximum a posteriori (MAP) predictions are shown for the uncertain nodes (shown as octagonal icons). CN, China; IT, Italy, EU, Europe.

The analysis of lineage B showed that only 2 nodes remained undetermined between Europe and China (Supplementary Fig. 4). The visualization (Fig. 5) suggested several introductions from China to Italy starting with the end of February. A single cluster corresponding to the previously described cluster#5 was observed, while the other strains apparently represent multiple independent introductions forming small groups of no more than 2 sequences. Two sporadic introductions from Europe were also observed. Unlike the ancestral reconstruction for B.1 lineage, this scenario was different as the migratory flows seem to stop in Italy without further spread.

The analysis conducted among European countries (Supplementary Fig. 5), highlighted the same ancestral scenario but did not show any introduction from Europe.

## Discussion

The present study shows that a few different SARS-CoV-2 lineages (B, B.1 and B.1.1, which largely correspond to clades 19A, 20A and 20B) were the most prevalent in Italy since the beginning of the pandemic, accounting for more than 93.8% of the 465 isolates in the dataset considered. This is in accordance with previous studies of the genomic epidemiology of SARS-CoV-2 in Italy performed by our and other Italian research teams^6,9,10^. Nevertheless, we observed important differences in the distribution of such lineages and clades both in space and in time. Several regions, mostly in Northern Italy, showed a high prevalence of B.1 lineage (clade 20A), while other regions, predominantly in Central/Southern Italy, were characterized by a higher prevalence of B.1.1 (clade 20B). Two regions presented a unique scenario with one highly predominant lineage: Veneto, in North-Eastern Italy, where a high prevalence of lineage B (19A clade) was observed in the early hit area of Padua and Piedmont, in North-Western Italy, where lineage B.1.1.1 (clade 20D) was highly prevalent (73%) as opposed to no cases in the other regions.

Lineages B and B.1, as well as clades 19A and 20A, were largely prevalent at the beginning of the epidemic between January and the first half of March, while other lineages (B.1.1, B.1.1.1) and clades (20B, 20C and 20D) were more prevalent later in the pandemic. The substitution of distinct SARS-CoV-2 lineages over time was confirmed by the estimation of the main lineages tMRCAs, suggesting an early spread in Italy of lineages B and B.1 in the second half of January 2020, followed later by the emergence of other B.1 descendants (B.1.1 and B.1.1.1) between late February and March. These results correlate with the consolidated epidemiological data showing that the first introduction of imported COVID-19 cases in Italy occurred at the end of January 2020, after the identification of two Chinese tourists in Rome infected by lineage B SARS-CoV-2, apparently without further spread. The first autochthonous cases of COVID-19 in Italy were documented several weeks later (21 February) when the first Italian transmissions without obvious connections with China were described in Lombardy (Codogno and in other centres of the Lodi’s area), where lineage B1 was prevalent, and Veneto (the province of Padua), where dominated lineage B at the beginning. On the contrary, the earliest cases in Central-Southern regions were reported a few days later (in Emilia Romagna and Tuscany on February 24 and in Sicily, Abruzzo, Marche and Apulia on February 26), while the number of cases remained relatively low until at least the second half of March (https://www.epicentro.iss.it/coronavirus/sars-cov-2-dashboard).

The analysis by ancestral character reconstruction of the Italian dataset, assigning each taxon to the region where it was sampled and the main viral lineage to which it belongs, showed two distinct patterns of dispersion of SARS-CoV-2. The first pathway concerns lineage B which was introduced to Veneto giving rise to a cluster that apparently disappeared in that region within the first half of March. The second pattern involved lineage B.1 which seems to have entered Lombardy and spread from there to other Italian regions, mainly in Central (i.e. Marche, Abruzzo) and Northern (i.e. Emilia Romagna, Veneto) Italy. This observation is in agreement with the epidemiological data showing the effective suppression of the SARS-CoV-2 outbreak in Veneto in the early times of the epidemic by a highly effective comprehensive testing and tracing approach and local lockdowns^11^. Central Italy (Abruzzo) seems to represent another important centre of dispersion of the lineage B.1.1 (descendant from B.1) mainly to South in mid-March. The introduction of the SARS-CoV-2 lineage B.1.1.1 occurred in Piedmont in the second half of March and apparently did not spread further.

The cluster analysis performed on the ML tree of the global dataset showed few and small (≤ 11 isolates) Italian clusters, including 17.2% of total Italian strains, while the majority of them were intermixed in the whole tree, frequently near the clades’ root (in particular for clades B.1 and B.1.1). This observation is in contrast with data from other European countries (i.e. Spain, Scotland or UK) describing from hundreds to thousands of phylogenetic clusters. This may be related to several reasons, including the poor sampling in the early stages of the Italian outbreak and the low variability of the virus. Nevertheless, Italy was one of the earliest European countries involved in the pandemic, thus the position of Italian strains near to the root of the tree is not surprising and highlights the central role played by Italy in the early spread of the epidemic. Moreover, most of the earliest clusters, dating before the implementation of the Italian national lockdown (2020-03-11), were frequently “mixed” or singletons, including international isolates, while clusters dating after the lockdown were mainly pure Italian. This could be related to the fact that the social distancing measures “froze” the transmission chain and in turn shut off viral evolution. The blockade or limitation of international travelling likely contributed to halt virus spread.

These observations were more deeply investigated by reconstructing the ancestral scenario from the ML trees obtained with the International data set which, similar to that obtained with Italian data set, showed two well defined phylogeographic patterns. The first pathway is that of lineage B showing a large number of independent introductions indicating multiple importation events, most probably from China (or elsewhere in Asia) to Italy as well as to other European countries, even if sporadic introductions to Italy from other European countries could not be excluded. The second phylogeographic scenario involving lineage B.1, showed initially only a few introductions from Asia to Italy and Europe (more precisely defined as Germany in the more detailed country based analysis)^1,12^, of small clusters corresponding to ancestral B.1/19A isolates, characterized by the substitution D614G in the spike protein in the absence of the P314L in the RdRp. A second introduction to Italy corresponded to the largest B.1 cluster characterized by all the substitutions typical of 20A clade giving rise to smaller clusters dispersed to other European and Asian countries and to a further large Italian cluster, corresponding to the lineage B.1.1 which was then dispersed to Italy and to Europe at multiple times. The biggest European cluster descending from B.1.1, corresponding to the lineage B.1.1.1, spread to Italy only in the second half of March. The phylogeographic analysis based on the sampling countries suggested the important roles played from several other European countries, in particular after the second week of March.

The major limitations of this study are intrinsic to the application of the phylogeographical approach to SARS-CoV-2 due to the relatively low evolutionary rate of SARS-CoV-2 in comparison with other RNA viruses, the limited number of sequences, available at the time of the study was performed, possibly affected by sampling bias for the characters underrepresented in several countries, including Italy, in the early epidemic and the rapid dissemination of the infection^3^. Furthermore, a frequent homoplasy^13,14^, that affects multiple protein sites of the viral genome, and the founder effect played a dominant role in the early evolution of the virus^15,16^. For these reasons, a limited number of studies on the SARS-CoV-2 phylogeography have been published, based on maximum parsimony^17^, maximum likelihood and Bayesian framework^3,18^ or phylogenetic network^19^. These studies analysed a limited number of genomes available at the time in the short interval elapsed since the origin of the virus^20^ while others highlighted the importance of including travel-related information in the analysis^18^.

These limitations could be overcome by adding more sequences and increasing the signal, which would allow to reduce biases and uncertainties. However, classical Bayesian phylogenetic methods, allowing the joint estimation of tree topology with evolutionary parameters and the character state, is very computationally demanding and time consuming; on the other hand, ML, based on ancestral character reconstruction, can be performed in large trees with thousands of tips in a relatively short time^21^. Another limitation of the study is that travel-related information was not available for all cases. Nevertheless, the absence of international travel among those for whom the information was available is probably due to a low and scattered sampling density restricted mainly to symptomatic patients and a prevalent circulation of the virus in small communities rather than in large cities, during the first phase of the epidemic in Italy. It therefore emphasizes the importance of phylogeographic reconstruction in attempting to formulate hypotheses on the possible flows of the virus in the international context.

In conclusion, a possible scenario was reconstructed by employing an ancestral character method allowing the analysis of a large amount of data. Based on our reconstruction, initial multiple sporadic introductions of B lineage to Italy occurred at least since the second half of January 2020 and remained relatively confined. Subsequently, in the month of February the D614G mutant entered in North Italy rapidly spreading to the rest of Italy and Europe, determining a different epidemiological profile of the Italian epidemic since then sustained only by B.1 lineage and his descendants. B.1.1 apparently emerged from the Italian B.1 cluster, suggesting a local evolution, lineage B.1.1.1 most probably emerged from B.1.1 in other European countries, and was introduced in Piedmont after the Italian national lockdown. Overall, our data suggests a central role of Italy in the exporting of some viral lineages at the beginning of the European epidemic, while subsequently, after mid-March, it was an importing centre from other European countries. Future studies, employing all the isolates collected in the early phases of the epidemic, many of which became available only after this study began, could provide more information about the origin of the pandemic spread in Italy and Europe.

The introduction in Italy of the D614G variant with a greater transmissibility and its hidden circulation for weeks before the detection of the first cases in Italy could be responsible for the rapid spread of the epidemic in Northern Italy followed by spread to other Italian regions and possibly to the rest of Europe, similar to what was observed for lineage B.1.7.7, firstly predominating in UK and, subsequently, in many other European (and extra-European) countries (eCDC, rapid risk assessment, 15 February 2021).

## Materials and methods

### Specimen collection

Sequence and epidemiological data were collected at the centres participating to the collaborative group SCIRE (SARS-CoV-2 Italian Research Enterprise), established at the beginning of the pandemic. SARS-CoV-2 RNA positive samples were collected between 24th February to 18th June 2020 from the respiratory tract of individuals who were either hospitalized or tested within screening programs. Samples were collected in most Italian regions, including Apulia, Campania, Emilia Romagna, Lazio, Liguria, Lombardy, Marche, Piedmont, Sardinia, Sicily, Tuscany, Umbria and Veneto. All participants gave the written informed consent to the storage of their anonymised data in a protected database. All of the data used in this study were previously anonymized as required by the Italian Data Protection Code (Legislative Decree 196/2003) and the general authorizations issued by the Data Protection Authority. The study was approved by the Ethical Committee of the Sacco Hospital (Prot N. 0,012,266) and conducted in compliance with Good Clinical Practice guidelines and the Declaration of Helsinki.

### Virus genome sequencing

SARS-CoV-2 RNA was extracted using the Kit QIAsymphony DSP Virus/Pathogen Midi kit on the QIAsymphony automated platform (QIAGEN, Hilden, Germany) (n = 11), the NucleoMag 96 Virus (Macherey–Nagel, Dueren, Germany) on automated KingFisher ml Magnetic Particle Processors (Thermo Fisher Scientific, Waltham, MA, USA) (n = 44) and manually with QIAamp Viral RNA Mini Kit (QIAGEN, Hilden, Germany) (n = 137). Full genome sequences were obtained with different protocols, by amplifying 26 fragments as previously described (n = 137)^10^ or by Ion AmpliSeq SARS-CoV-2 Research Panel (Thermo Fisher Scientific, Waltham, Massachusetts, USA) (n = 11) or by CleanPlex SARS-CoV-2 Panel (Paragon Genomics Inc, Hayward, CA, USA) (n = 44). Sequencing was performed on Illumina Miseq platform for all samples except for 11 that were sequenced with Ion GeneStudioS5 System instrument. The results were mapped and aligned to the reference genome obtained from GISAID (https://www.gisaid.org/, accession ID: EPI_ISL_406800) using Geneious Prime software v. 9.1.5 (Biomatters, Auckland, New Zealand) (http://www.geneious.com) or Torrent Suite v. 5.10.1 (Euformatics Oy, Espoo, Finland) or BWA-mem and rescued using Samtools alignment/Map (Hinxton, UK) (v. 1.9).

### SARS-CoV-2 data sets

A total of 254 genomes characterized by SCIRE group since the beginning of the collaboration (192 of which sequenced for this study), were combined with all the SARS-CoV-2 genomes collected between 29th January 2020 and 18th June 2020 available from Italy in the GISAID database (October 2020, Supplementary Table 1) to form the Italian data set (n = 465). Fourteen Chinese strains were added as outgroup obtaining a global dataset of 479 sequences.

To place the Italian sequences in the context of the international COVID-19 pandemic, an additional dataset was built including Chinese (n = 52) and European (n = 858) sequences collected in the same period. Due to the large amount of European sequences available, we included at least 2 strains per country/week (Supplementary Table 2 and Supplementary Fig. 6). Identical strains or those with more than 5% of gaps were excluded. For countries with a limited number of sequences, all strains were included. Only sequences reporting a certain sample collection were included. Consequently, the final dataset encompassed 1,375 sequences. SARS-CoV-2 sequences were aligned using MAFFT (https://mafft.cbrc.jp/alignment/server/) and the alignment was manually cropped using BioEdit v. 7.2.6.1 (https://bioedit.software.informer.com/).

### Genetic distance

The MEGA X program was used to evaluate the genetic distance between and within Italian strains on full length genome, with variance estimation performed using 1,000 bootstrap replicates. Amino acid changes were evaluated using MN908947 as the reference sequence (https://www.megasoftware.net/).

### Phylogenetic analysis

SARS-CoV-2 sequences were classified using the Pangolin COVID-19 Lineage Assigner tool v. 2.3.2 (last access 15 April 2021, https://pangolin.cog-uk.io/) and Nextclade v. 0.14.1 (https://clades.nextstrain.org/). The maximum likelihood trees of the two data sets were estimated using IQ-TREE v. 1.6.12 (http://www.iqtree.org/), using the GTR + F + R4 (General time reversible + empirical base frequencies + four number of categories) model selected by the program and 1,000 parametric bootstrap replicates for nodes support. Phylogenetic dating was obtained by the least squares dating method (LSD2) implemented in IQ-TREE with 100 replicates to obtain confidence intervals in node ages^22^. The genome sequence hCoV-19/Wuhan/WH04/2020|EPI_ISL_406801|2020-01-05 was used as an outgroup, as it falls in a basal position with respect to the tree and it results within a reasonable estimate of the time of emergence (time to the most recent common ancestor, tMRCA). Italian clusters (including more than 2 sequences) were identified in the ML tree by Cluster Picker v.1.2.3 using 80% bootstrap support and a mean genetic distance of less than 0.3% as thresholds. Epidemiological characteristics of the identified clusters were further investigated using Cluster Matcher v.1.2 ^23^ which allows the identification of clusters meeting given criteria. The Italian dataset was also analysed by BEAST v. 1.10 (https://beast.community/) in order to estimate the tMRCAs of the main clades. A previously estimated evolutionary rate of 8 × 10^−4^ substitutions/site^24^ and the selected substitution model (GTR + I + G) were used as priors. An exponential coalescent tree, with priors on population size and growth rate and an uncorrelated relaxed molecular clock model with an underlying lognormal distribution were chosen. Two runs of 150 million interactions were compared to assess convergence and then post-burnin samples pooled to summarize parameter estimates to obtain an effective samples sizes of at least 200. Finally, all trees were visualised in FigTree v. 1.4.4 (http://tree.bio.ed.ac.uk/software/figtree/).

### Phylogeographic analysis with PastML

The phylogeography was reconstructed from the time-scaled tree generated previously on the basis of annotated sampling location using PastML with maximum likelihood marginal posterior probabilities approximation (MPPA) and Felsenstein 1981 (F81) model options (https://pastml.pasteur.fr/). The PastML generated tree was visualized and edited using FigTree v.1.4.4 (http://tree.bio.ed.ac.uk/software/figtree/).

Each taxon was assigned to its sampling locations character, and we used PastML^21^ to reconstruct the ancestral character states and their changes along the trees. We used the MPPA as prediction method (standard settings) and added the character predicted by the joint reconstruction even if it was not selected by the Brier score (option-forced_joint). Additionally, we repeated the PastML analysis for the SARS-CoV-2 lineages. Phylogeographycal reconstruction was conducted using both Italian and European datasets.

In order to avoid ambiguities in the root reconstruction and given that lineages B and B.1 represent monophyletic groups we performed two independent ancestral state reconstructions: one for lineages B.1 and its descendent lineages (B.1.1 and B.1.1.1) and one for lineage B. The same outgroup was used for both analysis (EPI_ISL_406800).

## Supplementary Information

Supplementary Information.

## Data Availability

All consensus genomes are being submitted at the GISAID database (https://www.gisaid.org). All the files used for the analyses are available upon request.
