# Supplementary material for: Phylogeography and genomic epidemiology of SARS-CoV-2 in Italy and Europe with newly characterized Italian genomes between February-June 2020
Source: Sci Rep. 2022 Apr 6;12:5736. doi: 10.1038/s41598-022-09738-0 (PMC8986836; doi:10.1038/s41598-022-09738-0)
Supplement: Supplementary file 1 — Supplementary Information. [file 41598_2022_9738_MOESM1_ESM.docx]

**SUPPLEMENTARYINFORMATION**

| Accession ID | Sampling Date | |
| --- | --- | --- |
| EPI_ISL_569865 | 2020-04-08 |  |
| EPI_ISL_569868 | 2020-04-01 |  |
| EPI_ISL_569869 | 2020-03-31 |  |
| EPI_ISL_569870 | 2020-03-31 |  |
| EPI_ISL_569871 | 2020-03-31 |  |
| EPI_ISL_569872 | 2020-03-31 |  |
| EPI_ISL_569873 | 2020-03-31 |  |
| EPI_ISL_569874 | 2020-03-31 |  |
| EPI_ISL_569875 | 2020-03-31 |  |
| EPI_ISL_569876 | 2020-03-31 |  |
| EPI_ISL_569877 | 2020-03-31 |  |
| EPI_ISL_569878 | 2020-04-01 |  |
| EPI_ISL_569879 | 2020-04-01 |  |
| EPI_ISL_569880 | 2020-03-31 |  |
| EPI_ISL_569881 | 2020-04-01 |  |
| EPI_ISL_569882 | 2020-04-01 |  |
| EPI_ISL_569883 | 2020-04-08 |  |
| EPI_ISL_569884 | 2020-04-08 |  |
| EPI_ISL_569885 | 2020-04-08 |  |
| EPI_ISL_569886 | 2020-04-08 |  |
| EPI_ISL_525553 | 2020-03-16 |  |
| EPI_ISL_525556 | 2020-03-16 |  |
| EPI_ISL_525557 | 2020-03-17 |  |
| EPI_ISL_525558 | 2020-04-09 |  |
| EPI_ISL_525568 | 2020-04-11 |  |
| EPI_ISL_525573 | 2020-03-27 |  |
| EPI_ISL_525574 | 2020-04-08 |  |
| EPI_ISL_527380 | 2020-04-10 |  |
| EPI_ISL_412973 | 2020-02-20 |  |
| EPI_ISL_413489 | 2020-03-03 |  |
| EPI_ISL_417418 | 2020-03-01 |  |
| EPI_ISL_417419 | 2020-03-01 |  |
| EPI_ISL_417421 | 2020-03-01 |  |
| EPI_ISL_417423 | 2020-03-01 |  |
| EPI_ISL_417491 | 2020-03-03 |  |
| EPI_ISL_417921 | 2020-03-01 |  |
| EPI_ISL_417922 | 2020-02-28 |  |
| EPI_ISL_417923 | 2020-03-04 |  |
| EPI_ISL_418255 | 2020-03-14 |  |
| EPI_ISL_418256 | 2020-03-14 |  |
| EPI_ISL_418257 | 2020-03-17 |  |
| EPI_ISL_418258 | 2020-03-14 |  |
| EPI_ISL_418259 | 2020-03-14 |  |
| EPI_ISL_418260 | 2020-03-16 |  |
| EPI_ISL_418261 | 2020-03-17 |  |
| EPI_ISL_419254 | 2020-03-23 |  |
| EPI_ISL_419255 | 2020-03-23 |  |
| EPI_ISL_420563 | 2020-03-18 |  |
| EPI_ISL_420564 | 2020-03-19 |  |
| EPI_ISL_420565 | 2020-03-19 |  |
| EPI_ISL_420566 | 2020-03-19 |  |
| EPI_ISL_420567 | 2020-03-21 |  |
| EPI_ISL_420568 | 2020-03-23 |  |
| EPI_ISL_420569 | 2020-03-23 |  |
| EPI_ISL_420583 | 2020-03-23 |  |
| EPI_ISL_420592 | 2020-03-23 |  |
| EPI_ISL_422437 | 2020-03-25 |  |
| EPI_ISL_422438 | 2020-03-25 |  |
| EPI_ISL_424342 | 2020-03-07 |  |
| EPI_ISL_424343 | 2020-03-23 |  |
| EPI_ISL_424344 | 2020-03-04 |  |
| EPI_ISL_428853 | 2020-03-30 |  |
| EPI_ISL_428854 | 2020-03-30 |  |
| EPI_ISL_429226 | 2020-03-17 |  |
| EPI_ISL_429227 | 2020-03-17 |  |
| EPI_ISL_429228 | 2020-03-20 |  |
| EPI_ISL_429229 | 2020-03-21 |  |
| EPI_ISL_429230 | 2020-03-24 |  |
| EPI_ISL_429231 | 2020-03-24 |  |
| EPI_ISL_429232 | 2020-03-24 |  |
| EPI_ISL_429233 | 2020-03-24 |  |
| EPI_ISL_429234 | 2020-03-24 |  |
| EPI_ISL_429235 | 2020-03-24 |  |
| EPI_ISL_429236 | 2020-03-28 |  |
| EPI_ISL_435145 | 2020-03-24 |  |
| EPI_ISL_435146 | 2020-04-07 |  |
| EPI_ISL_435147 | 2020-04-08 |  |
| EPI_ISL_435148 | 2020-04-08 |  |
| EPI_ISL_435149 | 2020-04-08 |  |
| EPI_ISL_435150 | 2020-04-08 |  |
| EPI_ISL_435151 | 2020-04-08 |  |
| EPI_ISL_435152 | 2020-04-09 |  |
| EPI_ISL_435153 | 2020-04-09 |  |
| EPI_ISL_435154 | 2020-04-09 |  |
| EPI_ISL_435155 | 2020-04-09 |  |
| EPI_ISL_436718 | 2020-03-19 |  |
| EPI_ISL_436719 | 2020-03-20 |  |
| EPI_ISL_436720 | 2020-03-20 |  |
| EPI_ISL_436721 | 2020-03-20 |  |
| EPI_ISL_436722 | 2020-03-20 |  |
| EPI_ISL_436723 | 2020-03-20 |  |
| EPI_ISL_436724 | 2020-03-21 |  |
| EPI_ISL_436725 | 2020-04-27 |  |
| EPI_ISL_436726 | 2020-04-27 |  |
| EPI_ISL_436727 | 2020-04-27 |  |
| EPI_ISL_436728 | 2020-04-27 |  |
| EPI_ISL_436729 | 2020-04-27 |  |
| EPI_ISL_436730 | 2020-04-27 |  |
| EPI_ISL_436731 | 2020-04-26 |  |
| EPI_ISL_436732 | 2020-04-27 |  |
| EPI_ISL_451303 | 2020-03-04 |  |
| EPI_ISL_451304 | 2020-03-23 |  |
| EPI_ISL_451305 | 2020-03-23 |  |
| EPI_ISL_451306 | 2020-02-21 |  |
| EPI_ISL_451307 | 2020-02-21 |  |
| EPI_ISL_451308 | 2020-03-01 |  |
| EPI_ISL_451309 | 2020-03-01 |  |
| EPI_ISL_451961 | 2020-03-14 |  |
| EPI_ISL_451962 | 2020-03-20 |  |
| EPI_ISL_452181 | 2020-03-22 |  |
| EPI_ISL_452182 | 2020-03-23 |  |
| EPI_ISL_452183 | 2020-03-24 |  |
| EPI_ISL_452184 | 2020-03-24 |  |
| EPI_ISL_452185 | 2020-03-26 |  |
| EPI_ISL_452186 | 2020-03-28 |  |
| EPI_ISL_452187 | 2020-03-31 |  |
| EPI_ISL_452188 | 2020-03-31 |  |
| EPI_ISL_452189 | 2020-04-01 |  |
| EPI_ISL_452190 | 2020-04-03 |  |
| EPI_ISL_452191 | 2020-04-03 |  |
| EPI_ISL_454733 | 2020-03-01 |  |
| EPI_ISL_457699 | 2020-02-22 |  |
| EPI_ISL_457700 | 2020-03-06 |  |
| EPI_ISL_457721 | 2020-03-12 |  |
| EPI_ISL_457724 | 2020-03-15 |  |
| EPI_ISL_457728 | 2020-03-04 |  |
| EPI_ISL_457732 | 2020-03-14 |  |
| EPI_ISL_457736 | 2020-03-24 |  |
| EPI_ISL_457749 | 2020-02-27 |  |
| EPI_ISL_457825 | 2020-03-25 |  |
| EPI_ISL_457826 | 2020-03-08 |  |
| EPI_ISL_458084 | 2020-04-03 |  |
| EPI_ISL_458085 | 2020-04-12 |  |
| EPI_ISL_460079 | 2020-02-22 |  |
| EPI_ISL_460080 | 2020-02-28 |  |
| EPI_ISL_460081 | 2020-02-24 |  |
| EPI_ISL_460082 | 2020-02-26 |  |
| EPI_ISL_460083 | 2020-02-26 |  |
| EPI_ISL_460084 | 2020-02-29 |  |
| EPI_ISL_460085 | 2020-03-01 |  |
| EPI_ISL_460086 | 2020-02-24 |  |
| EPI_ISL_460087 | 2020-02-26 |  |
| EPI_ISL_460088 | 2020-02-26 |  |
| EPI_ISL_460089 | 2020-02-26 |  |
| EPI_ISL_460090 | 2020-02-28 |  |
| EPI_ISL_460091 | 2020-02-22 |  |
| EPI_ISL_460092 | 2020-02-28 |  |
| EPI_ISL_460093 | 2020-02-29 |  |
| EPI_ISL_460094 | 2020-02-25 |  |
| EPI_ISL_460095 | 2020-02-26 |  |
| EPI_ISL_468914 | 2020-03-18 |  |
| EPI_ISL_469016 | 2020-03-18 |  |
| EPI_ISL_469018 | 2020-03-19 |  |
| EPI_ISL_469019 | 2020-03-20 |  |
| EPI_ISL_469020 | 2020-03-19 |  |
| EPI_ISL_469021 | 2020-03-20 |  |
| EPI_ISL_469022 | 2020-03-20 |  |
| EPI_ISL_469023 | 2020-03-20 |  |
| EPI_ISL_477194 | 2020-03-16 |  |
| EPI_ISL_477195 | 2020-04-07 |  |
| EPI_ISL_477196 | 2020-04-06 |  |
| EPI_ISL_477197 | 2020-04-03 |  |
| EPI_ISL_477198 | 2020-03-20 |  |
| EPI_ISL_477199 | 2020-03-23 |  |
| EPI_ISL_477200 | 2020-03-23 |  |
| EPI_ISL_477201 | 2020-03-23 |  |
| EPI_ISL_477202 | 2020-03-20 |  |
| EPI_ISL_477203 | 2020-03-19 |  |
| EPI_ISL_477204 | 2020-05-30 |  |
| EPI_ISL_479616 | 2020-03-27 |  |
| EPI_ISL_479617 | 2020-03-27 |  |
| EPI_ISL_479618 | 2020-04-12 |  |
| EPI_ISL_479619 | 2020-04-12 |  |
| EPI_ISL_479790 | 2020-04-13 |  |
| EPI_ISL_479791 | 2020-03-08 |  |
| EPI_ISL_486646 | 2020-03-09 |  |
| EPI_ISL_486647 | 2020-03-02 |  |
| EPI_ISL_486648 | 2020-03-02 |  |
| EPI_ISL_486649 | 2020-03-13 |  |
| EPI_ISL_486650 | 2020-03-17 |  |
| EPI_ISL_486651 | 2020-03-24 |  |
| EPI_ISL_486652 | 2020-04-02 |  |
| EPI_ISL_486653 | 2020-03-20 |  |
| EPI_ISL_486654 | 2020-04-04 |  |
| EPI_ISL_486655 | 2020-04-02 |  |
| EPI_ISL_486656 | 2020-02-20 |  |
| EPI_ISL_486657 | 2020-02-26 |  |
| EPI_ISL_486658 | 2020-04-01 |  |
| EPI_ISL_486659 | 2020-02-22 |  |
| EPI_ISL_486660 | 2020-02-23 |  |
| EPI_ISL_486661 | 2020-03-02 |  |
| EPI_ISL_486662 | 2020-03-02 |  |
| EPI_ISL_486663 | 2020-03-06 |  |
| EPI_ISL_486664 | 2020-02-26 |  |
| EPI_ISL_486665 | 2020-03-03 |  |
| EPI_ISL_487276 | 2020-04-04 |  |
| EPI_ISL_492980 | 2020-03-18 |  |
| EPI_ISL_492981 | 2020-04-07 |  |
| EPI_ISL_492982 | 2020-04-07 |  |
| EPI_ISL_492983 | 2020-04-18 |  |
| EPI_ISL_492984 | 2020-03-11 |  |
| EPI_ISL_492985 | 2020-03-08 |  |
| EPI_ISL_492986 | 2020-03-10 |  |
| EPI_ISL_492987 | 2020-03-05 |  |
| EPI_ISL_493328 | 2020-03-15 |  |
| EPI_ISL_493329 | 2020-03-04 |  |
| EPI_ISL_493330 | 2020-05-10 |  |
| EPI_ISL_493331 | 2020-05-07 |  |
| EPI_ISL_493332 | 2020-03-28 |  |
| EPI_ISL_493333 | 2020-03-28 |  |
| EPI_ISL_496482 | 2020-03-17 |  |

**Supplementary Table 1. Accession IDs and sampling dates of the 211 Italian sequences retrieved from GISAID included in the dataset.**

| Accession ID | Sample date | |
| --- | --- | --- |
| EPI_ISL_416327 | 2020-01-28 |  |
| EPI_ISL_406800 | 2020-01-01 |  |
| EPI_ISL_412982 | 2020-02-07 |  |
| EPI_ISL_403929 | 2019-12-30 |  |
| EPI_ISL_41297 | 2020-01-29 |  |
| EPI_ISL_45129 | 2020-02-04 |  |
| EPI_ISL_416334 | 2020-02-06 |  |
| EPI_ISL_416386 | 2020-01-31 |  |
| EPI_ISL_417420 | 2020-03-23 |  |
| EPI_ISL_430743 | 2020-03-14 |  |
| EPI_ISL_430744 | 2020-03-14 |  |
| EPI_ISL_430745 | 2020-03-14 |  |
| EPI_ISL_430746 | 2020-03-14 |  |
| EPI_ISL_452330 | 2020-03-21 |  |
| EPI_ISL_452331 | 2020-03-12 |  |
| EPI_ISL_452337 | 2020-03-24 |  |
| EPI_ISL_452338 | 2020-03-19 |  |
| EPI_ISL_452339 | 2020-03-22 |  |
| EPI_ISL_452340 | 2020-03-19 |  |
| EPI_ISL_452341 | 2020-03-22 |  |
| EPI_ISL_452343 | 2020-03-16 |  |
| EPI_ISL_452345 | 2020-03-07 |  |
| EPI_ISL_452346 | 2020-03-19 |  |
| EPI_ISL_452347 | 2020-03-15 |  |
| EPI_ISL_452352 | 2020-03-05 |  |
| EPI_ISL_452353 | 2020-03-06 |  |
| EPI_ISL_452354 | 2020-03-07 |  |
| EPI_ISL_452356 | 2020-03-21 |  |
| EPI_ISL_962568 | 2020-02-01 |  |
| EPI_ISL_482577 | 2020-03-07 |  |
| EPI_ISL_482578 | 2020-03-12 |  |
| EPI_ISL_482581 | 2020-03-21 |  |
| EPI_ISL_482583 | 2020-03-22 |  |
| EPI_ISL_482585 | 2020-03-25 |  |
| EPI_ISL_482586 | 2020-03-25 |  |
| EPI_ISL_455690 | 2020-03-24 |  |
| EPI_ISL_455691 | 2020-03-24 |  |
| EPI_ISL_962530 | 2020-03-24 |  |
| EPI_ISL_962539 | 2020-03-11 |  |
| EPI_ISL_962535 | 2020-03-19 |  |
| EPI_ISL_962538 | 2020-03-12 |  |
| EPI_ISL_429089 | 2020-02-08 |  |
| EPI_ISL_431118 | 2020-03-13 |  |
| EPI_ISL_431780 | 2020-03-17 |  |
| EPI_ISL_431781 | 2020-03-19 |  |
| EPI_ISL_431782 | 2020-03-22 |  |
| EPI_ISL_430732 | 2020-01-29 |  |
| EPI_ISL_422425 | 2020-01-24 |  |
| EPI_ISL_429080 | 2020-02-05 |  |
| EPI_ISL_429081 | 2020-02-05 |  |
| EPI_ISL_451345 | 2020-01-24 |  |
| EPI_ISL_413593 | 2020-02-29 |  |
| EPI_ISL_414633 | 2020-03-04 |  |
| EPI_ISL_414640 | 2020-03-01 |  |
| EPI_ISL_416495 | 2020-03-10 |  |
| EPI_ISL_416510 | 2020-03-06 |  |
| EPI_ISL_417467 | 2020-03-15 |  |
| EPI_ISL_417481 | 2020-03-13 |  |
| EPI_ISL_417485 | 2020-03-02 |  |
| EPI_ISL_417721 | 2020-03-03 |  |
| EPI_ISL_418218 | 2020-02-21 |  |
| EPI_ISL_418220 | 2020-02-28 |  |
| EPI_ISL_419546 | 2020-03-15 |  |
| EPI_ISL_419562 | 2020-02-29 |  |
| EPI_ISL_419571 | 2020-03-06 |  |
| EPI_ISL_420138 | 2020-03-05 |  |
| EPI_ISL_420149 | 2020-03-09 |  |
| EPI_ISL_420312 | 2020-03-18 |  |
| EPI_ISL_424609 | 2020-03-29 |  |
| EPI_ISL_424655 | 2020-04-05 |  |
| EPI_ISL_428232 | 2020-03-18 |  |
| EPI_ISL_428930 | 2020-03-29 |  |
| EPI_ISL_429127 | 2020-03-20 |  |
| EPI_ISL_429277 | 2020-03-10 |  |
| EPI_ISL_429318 | 2020-03-16 |  |
| EPI_ISL_429357 | 2020-03-08 |  |
| EPI_ISL_429519 | 2020-03-22 |  |
| EPI_ISL_429722 | 2020-04-01 |  |
| EPI_ISL_429765 | 2020-03-22 |  |
| EPI_ISL_429968 | 2020-02-21 |  |
| EPI_ISL_435431 | 2020-03-25 |  |
| EPI_ISL_436321 | 2020-03-19 |  |
| EPI_ISL_436962 | 2020-04-02 |  |
| EPI_ISL_437253 | 2020-03-17 |  |
| EPI_ISL_437978 | 2020-03-11 |  |
| EPI_ISL_437981 | 2020-03-11 |  |
| EPI_ISL_447839 | 2020-03-24 |  |
| EPI_ISL_449792 | 2020-04-05 |  |
| EPI_ISL_450198 | 2020-01-28 |  |
| EPI_ISL_450288 | 2020-04-23 |  |
| EPI_ISL_450347 | 2020-03-25 |  |
| EPI_ISL_450519 | 2020-04-14 |  |
| EPI_ISL_450825 | 2020-04-27 |  |
| EPI_ISL_452016 | 2020-03-11 |  |
| EPI_ISL_452037 | 2020-04-14 |  |
| EPI_ISL_452038 | 2020-04-08 |  |
| EPI_ISL_452080 | 2020-04-16 |  |
| EPI_ISL_452235 | 2020-04-15 |  |
| EPI_ISL_454250 | 2020-04-06 |  |
| EPI_ISL_454266 | 2020-04-08 |  |
| EPI_ISL_454592 | 2020-03-05 |  |
| EPI_ISL_454606 | 2020-03-30 |  |
| EPI_ISL_455102 | 2020-04-27 |  |
| EPI_ISL_455105 | 2020-05-07 |  |
| EPI_ISL_455290 | 2020-04-06 |  |
| EPI_ISL_455445 | 2020-03-13 |  |
| EPI_ISL_455446 | 2020-03-13 |  |
| EPI_ISL_455472 | 2020-05-13 |  |
| EPI_ISL_455566 | 2020-03-30 |  |
| EPI_ISL_455567 | 2020-03-29 |  |
| EPI_ISL_455974 | 2020-05-11 |  |
| EPI_ISL_455976 | 2020-05-11 |  |
| EPI_ISL_459902 | 2020-05-19 |  |
| EPI_ISL_460605 | 2020-03-29 |  |
| EPI_ISL_460789 | 2020-04-10 |  |
| EPI_ISL_461209 | 2020-04-25 |  |
| EPI_ISL_462160 | 2020-03-30 |  |
| EPI_ISL_462435 | 2020-05-10 |  |
| EPI_ISL_463893 | 2020-05-25 |  |
| EPI_ISL_466921 | 2020-05-25 |  |
| EPI_ISL_467300 | 2020-04-08 |  |
| EPI_ISL_468958 | 2020-04-14 |  |
| EPI_ISL_470882 | 2020-03-09 |  |
| EPI_ISL_471175 | 2020-04-27 |  |
| EPI_ISL_471177 | 2020-05-04 |  |
| EPI_ISL_471428 | 2020-05-20 |  |
| EPI_ISL_471431 | 2020-05-28 |  |
| EPI_ISL_475103 | 2020-03-24 |  |
| EPI_ISL_475128 | 2020-03-12 |  |
| EPI_ISL_475154 | 2020-04-06 |  |
| EPI_ISL_475519 | 2020-05-14 |  |
| EPI_ISL_475529 | 2020-05-28 |  |
| EPI_ISL_475901 | 2020-04-07 |  |
| EPI_ISL_476109 | 2020-04-30 |  |
| EPI_ISL_476134 | 2020-04-17 |  |
| EPI_ISL_476836 | 2020-03-01 |  |
| EPI_ISL_477008 | 2020-04-21 |  |
| EPI_ISL_480300 | 2020-04-14 |  |
| EPI_ISL_480303 | 2020-03-30 |  |
| EPI_ISL_481208 | 2020-04-20 |  |
| EPI_ISL_481211 | 2020-04-28 |  |
| EPI_ISL_481212 | 2020-04-25 |  |
| EPI_ISL_481215 | 2020-05-25 |  |
| EPI_ISL_481216 | 2020-04-18 |  |
| EPI_ISL_481221 | 2020-04-24 |  |
| EPI_ISL_481519 | 2020-04-10 |  |
| EPI_ISL_468738 | 2020-03-13 |  |
| EPI_ISL_481536 | 2020-05-11 |  |
| EPI_ISL_481547 | 2020-04-12 |  |
| EPI_ISL_481565 | 2020-05-15 |  |
| EPI_ISL_481569 | 2020-03-16 |  |
| EPI_ISL_481570 | 2020-03-16 |  |
| EPI_ISL_481572 | 2020-04-17 |  |
| EPI_ISL_481582 | 2020-04-17 |  |
| EPI_ISL_481611 | 2020-04-21 |  |
| EPI_ISL_481616 | 2020-04-22 |  |
| EPI_ISL_481630 | 2020-04-28 |  |
| EPI_ISL_481635 | 2020-04-28 |  |
| EPI_ISL_481641 | 2020-03-29 |  |
| EPI_ISL_481663 | 2020-03-30 |  |
| EPI_ISL_481673 | 2020-04-04 |  |
| EPI_ISL_481677 | 2020-05-04 |  |
| EPI_ISL_481694 | 2020-05-05 |  |
| EPI_ISL_486432 | 2020-03-16 |  |
| EPI_ISL_486436 | 2020-03-25 |  |
| EPI_ISL_489709 | 2020-03-26 |  |
| EPI_ISL_490208 | 2020-05-11 |  |
| EPI_ISL_491062 | 2020-04-17 |  |
| EPI_ISL_491094 | 2020-03-20 |  |
| EPI_ISL_493351 | 2020-04-06 |  |
| EPI_ISL_501231 | 2020-04-25 |  |
| EPI_ISL_508704 | 2020-05-20 |  |
| EPI_ISL_508940 | 2020-03-09 |  |
| EPI_ISL_510129 | 2020-05-19 |  |
| EPI_ISL_510133 | 2020-05-04 |  |
| EPI_ISL_510811 | 2020-05-23 |  |
| EPI_ISL_510844 | 2020-05-20 |  |
| EPI_ISL_511702 | 2020-04-25 |  |
| EPI_ISL_513314 | 2020-03-06 |  |
| EPI_ISL_516645 | 2020-03-06 |  |
| EPI_ISL_525539 | 2020-03-16 |  |
| EPI_ISL_537874 | 2020-03-17 |  |
| EPI_ISL_539524 | 2020-03-15 |  |
| EPI_ISL_541335 | 2020-05-13 |  |
| EPI_ISL_548249 | 2020-04-14 |  |
| EPI_ISL_560587 | 2020-05-26 |  |
| EPI_ISL_560588 | 2020-05-28 |  |
| EPI_ISL_560590 | 2020-05-22 |  |
| EPI_ISL_568934 | 2020-04-16 |  |
| EPI_ISL_568959 | 2020-04-27 |  |
| EPI_ISL_568978 | 2020-05-13 |  |
| EPI_ISL_569311 | 2020-03-23 |  |
| EPI_ISL_569513 | 2020-04-04 |  |
| EPI_ISL_569589 | 2020-04-08 |  |
| EPI_ISL_577632 | 2020-05-16 |  |
| EPI_ISL_581917 | 2020-04-22 |  |
| EPI_ISL_582134 | 2020-02-15 |  |
| EPI_ISL_583636 | 2020-05-23 |  |
| EPI_ISL_583829 | 2020-04-09 |  |
| EPI_ISL_584080 | 2020-05-28 |  |
| EPI_ISL_590824 | 2020-03-09 |  |
| EPI_ISL_602471 | 2020-04-04 |  |
| EPI_ISL_603112 | 2020-04-20 |  |
| EPI_ISL_270101 | 2020-01-28 |  |
| EPI_ISL_270112 | 2020-01-30 |  |
| EPI_ISL_450200 | 2020-01-28 |  |
| EPI_ISL_270102 | 2020-01-29 |  |
| EPI_ISL_270104 | 2020-01-28 |  |
| EPI_ISL_270105 | 2020-01-28 |  |
| EPI_ISL_270106 | 2020-02-01 |  |
| EPI_ISL_270107 | 2020-02-01 |  |
| EPI_ISL_270109 | 2020-01-28 |  |
| EPI_ISL_270110 | 2020-02-07 |  |
| EPI_ISL_270111 | 2020-02-08 |  |
| EPI_ISL_270113 | 2020-02-01 |  |
| EPI_ISL_270108 | 2020-01-28 |  |
| EPI_ISL_450211 | 2020-02-01 |  |
| EPI_ISL_416144 | 2020-03-01 |  |
| EPI_ISL_417839 | 2020-03-16 |  |
| EPI_ISL_422636 | 2020-03-08 |  |
| EPI_ISL_428879 | 2020-03-19 |  |
| EPI_ISL_437896 | 2020-03-08 |  |
| EPI_ISL_491083 | 2020-04-28 |  |
| EPI_ISL_510427 | 2020-05-08 |  |
| EPI_ISL_538698 | 2020-03-24 |  |
| EPI_ISL_413556 | 2020-03-04 |  |
| EPI_ISL_415656 | 2020-03-12 |  |
| EPI_ISL_415977 | 2020-03-08 |  |
| EPI_ISL_415991 | 2020-03-09 |  |
| EPI_ISL_417265 | 2020-03-08 |  |
| EPI_ISL_417273 | 2020-03-06 |  |
| EPI_ISL_417307 | 2020-03-08 |  |
| EPI_ISL_417643 | 2020-03-18 |  |
| EPI_ISL_418087 | 2020-03-12 |  |
| EPI_ISL_418088 | 2020-03-12 |  |
| EPI_ISL_418089 | 2020-03-12 |  |
| EPI_ISL_418091 | 2020-03-12 |  |
| EPI_ISL_418094 | 2020-03-12 |  |
| EPI_ISL_420483 | 2020-03-18 |  |
| EPI_ISL_420484 | 2020-03-19 |  |
| EPI_ISL_420770 | 2020-03-27 |  |
| EPI_ISL_420913 | 2020-03-24 |  |
| EPI_ISL_420914 | 2020-03-12 |  |
| EPI_ISL_421824 | 2020-03-25 |  |
| EPI_ISL_421825 | 2020-03-25 |  |
| EPI_ISL_421831 | 2020-03-23 |  |
| EPI_ISL_422682 | 2020-03-19 |  |
| EPI_ISL_423175 | 2020-03-24 |  |
| EPI_ISL_423177 | 2020-03-24 |  |
| EPI_ISL_423301 | 2020-03-25 |  |
| EPI_ISL_423319 | 2020-03-26 |  |
| EPI_ISL_423495 | 2020-03-30 |  |
| EPI_ISL_423974 | 2020-03-20 |  |
| EPI_ISL_424004 | 2020-03-20 |  |
| EPI_ISL_424093 | 2020-03-12 |  |
| EPI_ISL_424094 | 2020-03-12 |  |
| EPI_ISL_424095 | 2020-03-10 |  |
| EPI_ISL_424097 | 2020-03-21 |  |
| EPI_ISL_424098 | 2020-03-21 |  |
| EPI_ISL_424121 | 2020-03-21 |  |
| EPI_ISL_424124 | 2020-03-21 |  |
| EPI_ISL_424165 | 2020-03-22 |  |
| EPI_ISL_424497 | 2020-03-20 |  |
| EPI_ISL_424510 | 2020-03-20 |  |
| EPI_ISL_425368 | 2020-03-16 |  |
| EPI_ISL_425400 | 2020-03-13 |  |
| EPI_ISL_425915 | 2020-03-23 |  |
| EPI_ISL_428236 | 2020-03-27 |  |
| EPI_ISL_428881 | 2020-03-16 |  |
| EPI_ISL_428889 | 2020-03-22 |  |
| EPI_ISL_428891 | 2020-03-25 |  |
| EPI_ISL_428910 | 2020-03-31 |  |
| EPI_ISL_429273 | 2020-03-10 |  |
| EPI_ISL_429523 | 2020-03-02 |  |
| EPI_ISL_432139 | 2020-04-02 |  |
| EPI_ISL_432147 | 2020-03-26 |  |
| EPI_ISL_432682 | 2020-04-07 |  |
| EPI_ISL_433036 | 2020-04-03 |  |
| EPI_ISL_433946 | 2020-04-12 |  |
| EPI_ISL_437284 | 2020-04-02 |  |
| EPI_ISL_437663 | 2020-03-02 |  |
| EPI_ISL_437879 | 2020-03-14 |  |
| EPI_ISL_437939 | 2020-04-06 |  |
| EPI_ISL_437970 | 2020-03-17 |  |
| EPI_ISL_438402 | 2020-03-28 |  |
| EPI_ISL_439501 | 2020-03-31 |  |
| EPI_ISL_439695 | 2020-04-08 |  |
| EPI_ISL_439703 | 2020-04-08 |  |
| EPI_ISL_439735 | 2020-04-08 |  |
| EPI_ISL_439749 | 2020-04-08 |  |
| EPI_ISL_439792 | 2020-04-12 |  |
| EPI_ISL_439854 | 2020-04-17 |  |
| EPI_ISL_440377 | 2020-03-17 |  |
| EPI_ISL_440446 | 2020-03-12 |  |
| EPI_ISL_440450 | 2020-03-12 |  |
| EPI_ISL_440543 | 2020-03-10 |  |
| EPI_ISL_440554 | 2020-03-04 |  |
| EPI_ISL_440623 | 2020-04-02 |  |
| EPI_ISL_440860 | 2020-04-16 |  |
| EPI_ISL_440937 | 2020-04-25 |  |
| EPI_ISL_441543 | 2020-04-18 |  |
| EPI_ISL_441592 | 2020-04-07 |  |
| EPI_ISL_441822 | 2020-04-03 |  |
| EPI_ISL_441857 | 2020-04-20 |  |
| EPI_ISL_441862 | 2020-04-21 |  |
| EPI_ISL_441877 | 2020-04-29 |  |
| EPI_ISL_443684 | 2020-04-13 |  |
| EPI_ISL_443734 | 2020-03-30 |  |
| EPI_ISL_444385 | 2020-04-27 |  |
| EPI_ISL_445468 | 2020-04-03 |  |
| EPI_ISL_445713 | 2020-04-11 |  |
| EPI_ISL_445884 | 2020-03-31 |  |
| EPI_ISL_445896 | 2020-03-31 |  |
| EPI_ISL_446394 | 2020-04-10 |  |
| EPI_ISL_446546 | 2020-04-13 |  |
| EPI_ISL_448825 | 2020-03-17 |  |
| EPI_ISL_449338 | 2020-03-25 |  |
| EPI_ISL_449361 | 2020-03-29 |  |
| EPI_ISL_449376 | 2020-03-28 |  |
| EPI_ISL_449378 | 2020-03-28 |  |
| EPI_ISL_449428 | 2020-03-27 |  |
| EPI_ISL_449433 | 2020-03-27 |  |
| EPI_ISL_449472 | 2020-03-26 |  |
| EPI_ISL_449521 | 2020-04-01 |  |
| EPI_ISL_449530 | 2020-03-31 |  |
| EPI_ISL_449558 | 2020-03-23 |  |
| EPI_ISL_449575 | 2020-04-02 |  |
| EPI_ISL_449583 | 2020-03-23 |  |
| EPI_ISL_449586 | 2020-03-24 |  |
| EPI_ISL_449594 | 2020-03-24 |  |
| EPI_ISL_449618 | 2020-04-29 |  |
| EPI_ISL_451725 | 2020-03-15 |  |
| EPI_ISL_451751 | 2020-03-25 |  |
| EPI_ISL_451856 | 2020-04-06 |  |
| EPI_ISL_451897 | 2020-04-07 |  |
| EPI_ISL_451899 | 2020-04-07 |  |
| EPI_ISL_452140 | 2020-04-03 |  |
| EPI_ISL_453198 | 2020-04-16 |  |
| EPI_ISL_453223 | 2020-04-10 |  |
| EPI_ISL_453236 | 2020-04-15 |  |
| EPI_ISL_453240 | 2020-04-04 |  |
| EPI_ISL_453247 | 2020-04-05 |  |
| EPI_ISL_453252 | 2020-04-07 |  |
| EPI_ISL_453264 | 2020-04-02 |  |
| EPI_ISL_453279 | 2020-04-03 |  |
| EPI_ISL_453303 | 2020-04-02 |  |
| EPI_ISL_453310 | 2020-04-03 |  |
| EPI_ISL_453330 | 2020-04-10 |  |
| EPI_ISL_453339 | 2020-04-11 |  |
| EPI_ISL_453349 | 2020-04-12 |  |
| EPI_ISL_453352 | 2020-04-12 |  |
| EPI_ISL_453358 | 2020-04-12 |  |
| EPI_ISL_453366 | 2020-04-05 |  |
| EPI_ISL_453372 | 2020-04-04 |  |
| EPI_ISL_453373 | 2020-04-04 |  |
| EPI_ISL_453383 | 2020-04-04 |  |
| EPI_ISL_453385 | 2020-04-04 |  |
| EPI_ISL_453393 | 2020-04-20 |  |
| EPI_ISL_453400 | 2020-04-04 |  |
| EPI_ISL_453419 | 2020-04-15 |  |
| EPI_ISL_454330 | 2020-03-13 |  |
| EPI_ISL_454787 | 2020-03-12 |  |
| EPI_ISL_454793 | 2020-03-31 |  |
| EPI_ISL_457307 | 2020-04-12 |  |
| EPI_ISL_458742 | 2020-04-20 |  |
| EPI_ISL_459126 | 2020-04-06 |  |
| EPI_ISL_459447 | 2020-04-27 |  |
| EPI_ISL_459540 | 2020-03-27 |  |
| EPI_ISL_462251 | 2020-04-07 |  |
| EPI_ISL_464494 | 2020-03-03 |  |
| EPI_ISL_464818 | 2020-03-11 |  |
| EPI_ISL_464886 | 2020-03-13 |  |
| EPI_ISL_464945 | 2020-03-12 |  |
| EPI_ISL_464946 | 2020-03-12 |  |
| EPI_ISL_465407 | 2020-04-20 |  |
| EPI_ISL_465415 | 2020-04-21 |  |
| EPI_ISL_465455 | 2020-04-27 |  |
| EPI_ISL_465458 | 2020-04-28 |  |
| EPI_ISL_465707 | 2020-04-28 |  |
| EPI_ISL_465709 | 2020-04-28 |  |
| EPI_ISL_465806 | 2020-03-11 |  |
| EPI_ISL_465945 | 2020-03-30 |  |
| EPI_ISL_465989 | 2020-03-27 |  |
| EPI_ISL_466001 | 2020-03-31 |  |
| EPI_ISL_466087 | 2020-04-03 |  |
| EPI_ISL_466194 | 2020-04-02 |  |
| EPI_ISL_466324 | 2020-04-09 |  |
| EPI_ISL_466984 | 2020-03-12 |  |
| EPI_ISL_469074 | 2020-03-09 |  |
| EPI_ISL_469540 | 2020-04-22 |  |
| EPI_ISL_472165 | 2020-03-15 |  |
| EPI_ISL_474229 | 2020-03-18 |  |
| EPI_ISL_474277 | 2020-04-04 |  |
| EPI_ISL_474591 | 2020-04-27 |  |
| EPI_ISL_475764 | 2020-03-25 |  |
| EPI_ISL_475833 | 2020-03-21 |  |
| EPI_ISL_475934 | 2020-04-06 |  |
| EPI_ISL_478508 | 2020-03-31 |  |
| EPI_ISL_483672 | 2020-04-14 |  |
| EPI_ISL_483673 | 2020-04-17 |  |
| EPI_ISL_483677 | 2020-04-21 |  |
| EPI_ISL_488050 | 2020-04-22 |  |
| EPI_ISL_488313 | 2020-04-21 |  |
| EPI_ISL_488440 | 2020-04-22 |  |
| EPI_ISL_489542 | 2020-04-22 |  |
| EPI_ISL_492216 | 2020-04-01 |  |
| EPI_ISL_492236 | 2020-04-18 |  |
| EPI_ISL_492287 | 2020-04-07 |  |
| EPI_ISL_492668 | 2020-04-12 |  |
| EPI_ISL_492846 | 2020-03-26 |  |
| EPI_ISL_499842 | 2020-03-14 |  |
| EPI_ISL_499889 | 2020-03-13 |  |
| EPI_ISL_524559 | 2020-03-29 |  |
| EPI_ISL_528276 | 2020-03-20 |  |
| EPI_ISL_529691 | 2020-04-02 |  |
| EPI_ISL_538091 | 2020-03-24 |  |
| EPI_ISL_574911 | 2020-03-19 |  |
| EPI_ISL_581469 | 2020-03-23 |  |
| EPI_ISL_583557 | 2020-03-30 |  |
| EPI_ISL_583574 | 2020-03-27 |  |
| EPI_ISL_583576 | 2020-03-22 |  |
| EPI_ISL_583854 | 2020-04-06 |  |
| EPI_ISL_583861 | 2020-04-11 |  |
| EPI_ISL_583865 | 2020-04-14 |  |
| EPI_ISL_583867 | 2020-04-20 |  |
| EPI_ISL_613541 | 2020-03-02 |  |
| EPI_ISL_622342 | 2020-04-20 |  |
| EPI_ISL_574945 | 2020-03-17 |  |
| EPI_ISL_412912 | 2020-02-25 |  |
| EPI_ISL_413569 | 2020-02-28 |  |
| EPI_ISL_413570 | 2020-02-28 |  |
| EPI_ISL_413647 | 2020-03-01 |  |
| EPI_ISL_414425 | 2020-03-03 |  |
| EPI_ISL_414429 | 2020-03-02 |  |
| EPI_ISL_414641 | 2020-03-05 |  |
| EPI_ISL_414644 | 2020-03-04 |  |
| EPI_ISL_415129 | 2020-02-29 |  |
| EPI_ISL_415157 | 2020-03-01 |  |
| EPI_ISL_415158 | 2020-03-01 |  |
| EPI_ISL_416142 | 2020-02-26 |  |
| EPI_ISL_417688 | 2020-03-01 |  |
| EPI_ISL_417840 | 2020-03-02 |  |
| EPI_ISL_418548 | 2020-03-06 |  |
| EPI_ISL_418584 | 2020-03-10 |  |
| EPI_ISL_420687 | 2020-03-28 |  |
| EPI_ISL_425753 | 2020-03-12 |  |
| EPI_ISL_426286 | 2020-03-30 |  |
| EPI_ISL_426891 | 2020-03-26 |  |
| EPI_ISL_427043 | 2020-03-18 |  |
| EPI_ISL_427317 | 2020-04-03 |  |
| EPI_ISL_428865 | 2020-03-11 |  |
| EPI_ISL_428866 | 2020-03-11 |  |
| EPI_ISL_429120 | 2020-03-11 |  |
| EPI_ISL_429721 | 2020-03-31 |  |
| EPI_ISL_429864 | 2020-03-22 |  |
| EPI_ISL_430081 | 2020-04-10 |  |
| EPI_ISL_430096 | 2020-04-14 |  |
| EPI_ISL_430109 | 2020-04-15 |  |
| EPI_ISL_434461 | 2020-03-29 |  |
| EPI_ISL_434572 | 2020-04-14 |  |
| EPI_ISL_434676 | 2020-04-20 |  |
| EPI_ISL_437090 | 2020-03-24 |  |
| EPI_ISL_437257 | 2020-03-20 |  |
| EPI_ISL_437333 | 2020-03-25 |  |
| EPI_ISL_437993 | 2020-02-27 |  |
| EPI_ISL_437995 | 2020-02-28 |  |
| EPI_ISL_438007 | 2020-03-05 |  |
| EPI_ISL_438019 | 2020-03-08 |  |
| EPI_ISL_438074 | 2020-03-20 |  |
| EPI_ISL_445238 | 2020-04-20 |  |
| EPI_ISL_446848 | 2020-04-16 |  |
| EPI_ISL_447641 | 2020-03-18 |  |
| EPI_ISL_447645 | 2020-03-09 |  |
| EPI_ISL_450295 | 2020-04-11 |  |
| EPI_ISL_450338 | 2020-04-11 |  |
| EPI_ISL_450520 | 2020-04-28 |  |
| EPI_ISL_451652 | 2020-04-30 |  |
| EPI_ISL_451973 | 2020-03-31 |  |
| EPI_ISL_539034 | 2020-03-31 |  |
| EPI_ISL_539556 | 2020-03-02 |  |
| EPI_ISL_451795 | 2020-03-30 |  |
| EPI_ISL_452097 | 2020-05-09 |  |
| EPI_ISL_454107 | 2020-04-23 |  |
| EPI_ISL_454191 | 2020-04-18 |  |
| EPI_ISL_454194 | 2020-04-18 |  |
| EPI_ISL_454215 | 2020-04-29 |  |
| EPI_ISL_454581 | 2020-03-18 |  |
| EPI_ISL_454595 | 2020-03-09 |  |
| EPI_ISL_454871 | 2020-03-04 |  |
| EPI_ISL_455100 | 2020-03-31 |  |
| EPI_ISL_455443 | 2020-04-01 |  |
| EPI_ISL_455447 | 2020-03-25 |  |
| EPI_ISL_462270 | 2020-05-06 |  |
| EPI_ISL_462434 | 2020-04-01 |  |
| EPI_ISL_462480 | 2020-05-13 |  |
| EPI_ISL_462753 | 2020-05-27 |  |
| EPI_ISL_462990 | 2020-04-29 |  |
| EPI_ISL_464184 | 2020-02-25 |  |
| EPI_ISL_465568 | 2020-05-01 |  |
| EPI_ISL_466906 | 2020-05-04 |  |
| EPI_ISL_466908 | 2020-05-07 |  |
| EPI_ISL_467775 | 2020-03-29 |  |
| EPI_ISL_468155 | 2020-04-18 |  |
| EPI_ISL_469058 | 2020-05-07 |  |
| EPI_ISL_469079 | 2020-03-05 |  |
| EPI_ISL_470196 | 2020-04-20 |  |
| EPI_ISL_471547 | 2020-03-11 |  |
| EPI_ISL_475115 | 2020-03-30 |  |
| EPI_ISL_476069 | 2020-03-30 |  |
| EPI_ISL_476071 | 2020-04-08 |  |
| EPI_ISL_476086 | 2020-05-18 |  |
| EPI_ISL_476096 | 2020-05-27 |  |
| EPI_ISL_476132 | 2020-04-18 |  |
| EPI_ISL_478385 | 2020-05-26 |  |
| EPI_ISL_480253 | 2020-05-01 |  |
| EPI_ISL_480302 | 2020-05-07 |  |
| EPI_ISL_480308 | 2020-05-11 |  |
| EPI_ISL_486418 | 2020-05-09 |  |
| EPI_ISL_486438 | 2020-05-28 |  |
| EPI_ISL_486817 | 2020-03-30 |  |
| EPI_ISL_487370 | 2020-04-02 |  |
| EPI_ISL_487377 | 2020-04-02 |  |
| EPI_ISL_491118 | 2020-04-01 |  |
| EPI_ISL_491174 | 2020-05-17 |  |
| EPI_ISL_491186 | 2020-05-20 |  |
| EPI_ISL_491291 | 2020-05-27 |  |
| EPI_ISL_492073 | 2020-04-18 |  |
| EPI_ISL_500086 | 2020-03-05 |  |
| EPI_ISL_500196 | 2020-04-20 |  |
| EPI_ISL_501233 | 2020-05-03 |  |
| EPI_ISL_501235 | 2020-05-11 |  |
| EPI_ISL_501236 | 2020-05-13 |  |
| EPI_ISL_501249 | 2020-04-22 |  |
| EPI_ISL_501250 | 2020-05-08 |  |
| EPI_ISL_501254 | 2020-05-22 |  |
| EPI_ISL_501255 | 2020-05-25 |  |
| EPI_ISL_501257 | 2020-05-27 |  |
| EPI_ISL_507232 | 2020-05-02 |  |
| EPI_ISL_510516 | 2020-05-21 |  |
| EPI_ISL_510958 | 2020-03-14 |  |
| EPI_ISL_511035 | 2020-03-14 |  |
| EPI_ISL_511074 | 2020-03-20 |  |
| EPI_ISL_511390 | 2020-03-24 |  |
| EPI_ISL_511426 | 2020-03-16 |  |
| EPI_ISL_511683 | 2020-05-06 |  |
| EPI_ISL_511684 | 2020-05-06 |  |
| EPI_ISL_523126 | 2020-05-30 |  |
| EPI_ISL_523357 | 2020-05-09 |  |
| EPI_ISL_523998 | 2020-05-04 |  |
| EPI_ISL_532236 | 2020-05-13 |  |
| EPI_ISL_541332 | 2020-05-24 |  |
| EPI_ISL_541333 | 2020-05-23 |  |
| EPI_ISL_548956 | 2020-05-12 |  |
| EPI_ISL_560574 | 2020-03-25 |  |
| EPI_ISL_568950 | 2020-04-21 |  |
| EPI_ISL_569736 | 2020-05-08 |  |
| EPI_ISL_569746 | 2020-05-19 |  |
| EPI_ISL_569763 | 2020-05-13 |  |
| EPI_ISL_583572 | 2020-03-19 |  |
| EPI_ISL_583613 | 2020-03-27 |  |
| EPI_ISL_583620 | 2020-03-29 |  |
| EPI_ISL_583674 | 2020-04-21 |  |
| EPI_ISL_590834 | 2020-05-04 |  |
| EPI_ISL_596346 | 2020-05-27 |  |
| EPI_ISL_596645 | 2020-04-03 |  |
| EPI_ISL_596646 | 2020-03-29 |  |
| EPI_ISL_596648 | 2020-03-24 |  |
| EPI_ISL_596649 | 2020-03-30 |  |
| EPI_ISL_407079 | 2020-01-29 |  |
| EPI_ISL_408430 | 2020-01-29 |  |
| EPI_ISL_408431 | 2020-01-29 |  |
| EPI_ISL_410486 | 2020-02-08 |  |
| EPI_ISL_414041 | 2020-02-08 |  |
| EPI_ISL_414042 | 2020-02-08 |  |
| EPI_ISL_414487 | 2020-03-04 |  |
| EPI_ISL_422617 | 2020-04-02 |  |
| EPI_ISL_437300 | 2020-04-14 |  |
| EPI_ISL_455324 | 2020-03-03 |  |
| EPI_ISL_486426 | 2020-03-22 |  |
| EPI_ISL_499490 | 2020-03-22 |  |
| EPI_ISL_467778 | 2020-04-27 |  |
| EPI_ISL_468136 | 2020-03-22 |  |
| EPI_ISL_468137 | 2020-03-22 |  |
| EPI_ISL_468138 | 2020-03-25 |  |
| EPI_ISL_468142 | 2020-03-25 |  |
| EPI_ISL_468278 | 2020-04-28 |  |
| EPI_ISL_468656 | 2020-04-09 |  |
| EPI_ISL_468729 | 2020-03-09 |  |
| EPI_ISL_471172 | 2020-04-17 |  |
| EPI_ISL_471530 | 2020-03-10 |  |
| EPI_ISL_473081 | 2020-05-12 |  |
| EPI_ISL_474814 | 2020-05-14 |  |
| EPI_ISL_475124 | 2020-04-09 |  |
| EPI_ISL_475892 | 2020-03-31 |  |
| EPI_ISL_476099 | 2020-05-28 |  |
| EPI_ISL_476847 | 2020-04-12 |  |
| EPI_ISL_476997 | 2020-05-02 |  |
| EPI_ISL_477617 | 2020-03-20 |  |
| EPI_ISL_480307 | 2020-03-30 |  |
| EPI_ISL_480309 | 2020-04-19 |  |
| EPI_ISL_480310 | 2020-03-10 |  |
| EPI_ISL_481228 | 2020-04-18 |  |
| EPI_ISL_481549 | 2020-03-13 |  |
| EPI_ISL_486834 | 2020-05-25 |  |
| EPI_ISL_491055 | 2020-04-11 |  |
| EPI_ISL_491072 | 2020-04-22 |  |
| EPI_ISL_491085 | 2020-04-05 |  |
| EPI_ISL_491091 | 2020-05-22 |  |
| EPI_ISL_491119 | 2020-04-08 |  |
| EPI_ISL_491211 | 2020-05-28 |  |
| EPI_ISL_500339 | 2020-04-20 |  |
| EPI_ISL_508934 | 2020-03-01 |  |
| EPI_ISL_508966 | 2020-04-02 |  |
| EPI_ISL_511528 | 2020-03-30 |  |
| EPI_ISL_523949 | 2020-03-07 |  |
| EPI_ISL_526219 | 2020-04-30 |  |
| EPI_ISL_526220 | 2020-05-11 |  |
| EPI_ISL_526223 | 2020-05-25 |  |
| EPI_ISL_526236 | 2020-05-08 |  |
| EPI_ISL_527947 | 2020-03-06 |  |
| EPI_ISL_537361 | 2020-04-13 |  |
| EPI_ISL_537805 | 2020-03-23 |  |
| EPI_ISL_538122 | 2020-04-07 |  |
| EPI_ISL_539533 | 2020-05-13 |  |
| EPI_ISL_541032 | 2020-05-02 |  |
| EPI_ISL_568925 | 2020-04-15 |  |
| EPI_ISL_569606 | 2020-04-10 |  |
| EPI_ISL_574959 | 2020-03-16 |  |
| EPI_ISL_574978 | 2020-03-16 |  |
| EPI_ISL_581468 | 2020-04-23 |  |
| EPI_ISL_581691 | 2020-04-09 |  |
| EPI_ISL_581695 | 2020-04-10 |  |
| EPI_ISL_581763 | 2020-03-26 |  |
| EPI_ISL_581920 | 2020-04-24 |  |
| EPI_ISL_583589 | 2020-04-01 |  |
| EPI_ISL_583687 | 2020-04-13 |  |
| EPI_ISL_583716 | 2020-05-04 |  |
| EPI_ISL_583719 | 2020-05-06 |  |
| EPI_ISL_583723 | 2020-05-13 |  |
| EPI_ISL_584073 | 2020-05-10 |  |
| EPI_ISL_602483 | 2020-04-12 |  |
| EPI_ISL_467222 | 2020-04-01 |  |
| EPI_ISL_577629 | 2020-04-02 |  |
| EPI_ISL_406596 | 2020-01-23 |  |
| EPI_ISL_412116 | 2020-02-09 |  |
| EPI_ISL_413019 | 2020-02-26 |  |
| EPI_ISL_414013 | 2020-03-01 |  |
| EPI_ISL_414468 | 2020-03-06 |  |
| EPI_ISL_414529 | 2020-03-04 |  |
| EPI_ISL_414531 | 2020-03-04 |  |
| EPI_ISL_415139 | 2020-03-01 |  |
| EPI_ISL_415484 | 2020-03-13 |  |
| EPI_ISL_415512 | 2020-03-09 |  |
| EPI_ISL_416503 | 2020-03-01 |  |
| EPI_ISL_416507 | 2020-03-05 |  |
| EPI_ISL_417009 | 2020-03-07 |  |
| EPI_ISL_417011 | 2020-03-07 |  |
| EPI_ISL_417017 | 2020-03-14 |  |
| EPI_ISL_417025 | 2020-03-15 |  |
| EPI_ISL_417213 | 2020-03-01 |  |
| EPI_ISL_417590 | 2020-03-18 |  |
| EPI_ISL_417653 | 2020-03-18 |  |
| EPI_ISL_417661 | 2020-03-14 |  |
| EPI_ISL_417668 | 2020-03-15 |  |
| EPI_ISL_417670 | 2020-03-15 |  |
| EPI_ISL_417765 | 2020-02-27 |  |
| EPI_ISL_417801 | 2020-03-13 |  |
| EPI_ISL_417952 | 2020-03-09 |  |
| EPI_ISL_418021 | 2020-03-16 |  |
| EPI_ISL_418025 | 2020-03-17 |  |
| EPI_ISL_418287 | 2020-03-16 |  |
| EPI_ISL_418432 | 2020-03-18 |  |
| EPI_ISL_418805 | 2020-03-06 |  |
| EPI_ISL_419552 | 2020-03-16 |  |
| EPI_ISL_419658 | 2020-03-06 |  |
| EPI_ISL_420274 | 2020-03-22 |  |
| EPI_ISL_420369 | 2020-03-19 |  |
| EPI_ISL_420422 | 2020-03-25 |  |
| EPI_ISL_422114 | 2020-03-25 |  |
| EPI_ISL_422580 | 2020-03-30 |  |
| EPI_ISL_422604 | 2020-04-01 |  |
| EPI_ISL_422653 | 2020-03-18 |  |
| EPI_ISL_422673 | 2020-03-17 |  |
| EPI_ISL_422685 | 2020-03-19 |  |
| EPI_ISL_422725 | 2020-03-23 |  |
| EPI_ISL_422726 | 2020-03-23 |  |
| EPI_ISL_422743 | 2020-03-25 |  |
| EPI_ISL_422776 | 2020-03-28 |  |
| EPI_ISL_422845 | 2020-03-12 |  |
| EPI_ISL_422905 | 2020-03-13 |  |
| EPI_ISL_422807 | 2020-03-08 |  |
| EPI_ISL_423465 | 2020-03-30 |  |
| EPI_ISL_424503 | 2020-03-20 |  |
| EPI_ISL_424551 | 2020-03-25 |  |
| EPI_ISL_424556 | 2020-03-29 |  |
| EPI_ISL_424564 | 2020-03-27 |  |
| EPI_ISL_417864 | 2020-03-15 |  |
| EPI_ISL_425284 | 2020-03-18 |  |
| EPI_ISL_425668 | 2020-03-14 |  |
| EPI_ISL_432686 | 2020-04-07 |  |
| EPI_ISL_433829 | 2020-04-08 |  |
| EPI_ISL_434381 | 2020-04-16 |  |
| EPI_ISL_434459 | 2020-03-29 |  |
| EPI_ISL_434462 | 2020-03-23 |  |
| EPI_ISL_434466 | 2020-04-04 |  |
| EPI_ISL_434481 | 2020-03-19 |  |
| EPI_ISL_434485 | 2020-03-12 |  |
| EPI_ISL_436260 | 2020-03-19 |  |
| EPI_ISL_437231 | 2020-03-25 |  |
| EPI_ISL_437243 | 2020-04-11 |  |
| EPI_ISL_420903 | 2020-03-14 |  |
| EPI_ISL_437272 | 2020-03-30 |  |
| EPI_ISL_437303 | 2020-04-17 |  |
| EPI_ISL_437359 | 2020-03-14 |  |
| EPI_ISL_437883 | 2020-03-15 |  |
| EPI_ISL_437888 | 2020-03-12 |  |
| EPI_ISL_437884 | 2020-03-16 |  |
| EPI_ISL_437895 | 2020-03-07 |  |
| EPI_ISL_437906 | 2020-03-10 |  |
| EPI_ISL_437909 | 2020-03-27 |  |
| EPI_ISL_437910 | 2020-04-01 |  |
| EPI_ISL_437967 | 2020-03-13 |  |
| EPI_ISL_438064 | 2020-03-18 |  |
| EPI_ISL_438083 | 2020-03-22 |  |
| EPI_ISL_438281 | 2020-03-29 |  |
| EPI_ISL_438531 | 2020-04-01 |  |
| EPI_ISL_440451 | 2020-03-11 |  |
| EPI_ISL_440471 | 2020-03-12 |  |
| EPI_ISL_441099 | 2020-04-03 |  |
| EPI_ISL_443666 | 2020-04-13 |  |
| EPI_ISL_445674 | 2020-04-06 |  |
| EPI_ISL_446526 | 2020-04-11 |  |
| EPI_ISL_449149 | 2020-04-16 |  |
| EPI_ISL_449355 | 2020-03-29 |  |
| EPI_ISL_451740 | 2020-03-25 |  |
| EPI_ISL_451758 | 2020-03-26 |  |
| EPI_ISL_451791 | 2020-03-25 |  |
| EPI_ISL_451818 | 2020-03-31 |  |
| EPI_ISL_451942 | 2020-03-30 |  |
| EPI_ISL_452180 | 2020-03-20 |  |
| EPI_ISL_452383 | 2020-03-16 |  |
| EPI_ISL_452390 | 2020-03-17 |  |
| EPI_ISL_452462 | 2020-03-12 |  |
| EPI_ISL_452519 | 2020-03-15 |  |
| EPI_ISL_452601 | 2020-03-27 |  |
| EPI_ISL_452875 | 2020-05-14 |  |
| EPI_ISL_453003 | 2020-05-18 |  |
| EPI_ISL_453139 | 2020-04-27 |  |
| EPI_ISL_453890 | 2020-03-28 |  |
| EPI_ISL_453937 | 2020-03-30 |  |
| EPI_ISL_453992 | 2020-04-05 |  |
| EPI_ISL_453996 | 2020-04-05 |  |
| EPI_ISL_454003 | 2020-03-09 |  |
| EPI_ISL_454222 | 2020-03-11 |  |
| EPI_ISL_454257 | 2020-04-07 |  |
| EPI_ISL_454258 | 2020-04-07 |  |
| EPI_ISL_454259 | 2020-04-07 |  |
| EPI_ISL_454319 | 2020-03-28 |  |
| EPI_ISL_455140 | 2020-04-03 |  |
| EPI_ISL_455153 | 2020-04-06 |  |
| EPI_ISL_455160 | 2020-04-07 |  |
| EPI_ISL_455297 | 2020-04-07 |  |
| EPI_ISL_456690 | 2020-05-18 |  |
| EPI_ISL_456807 | 2020-05-06 |  |
| EPI_ISL_457480 | 2020-05-05 |  |
| EPI_ISL_457566 | 2020-05-13 |  |
| EPI_ISL_458221 | 2020-03-30 |  |
| EPI_ISL_458232 | 2020-03-20 |  |
| EPI_ISL_458506 | 2020-05-01 |  |
| EPI_ISL_459964 | 2020-03-28 |  |
| EPI_ISL_460783 | 2020-03-26 |  |
| EPI_ISL_461124 | 2020-04-13 |  |
| EPI_ISL_461315 | 2020-05-04 |  |
| EPI_ISL_461584 | 2020-05-28 |  |
| EPI_ISL_461844 | 2020-04-29 |  |
| EPI_ISL_462159 | 2020-03-30 |  |
| EPI_ISL_462183 | 2020-03-30 |  |
| EPI_ISL_462185 | 2020-03-30 |  |
| EPI_ISL_462209 | 2020-03-22 |  |
| EPI_ISL_462215 | 2020-03-31 |  |
| EPI_ISL_462269 | 2020-04-09 |  |
| EPI_ISL_464079 | 2020-04-06 |  |
| EPI_ISL_464089 | 2020-04-09 |  |
| EPI_ISL_464168 | 2020-02-09 |  |
| EPI_ISL_464170 | 2020-02-13 |  |
| EPI_ISL_464172 | 2020-02-14 |  |
| EPI_ISL_464175 | 2020-02-16 |  |
| EPI_ISL_464176 | 2020-02-16 |  |
| EPI_ISL_464177 | 2020-02-16 |  |
| EPI_ISL_464178 | 2020-02-18 |  |
| EPI_ISL_464179 | 2020-02-19 |  |
| EPI_ISL_464180 | 2020-02-20 |  |
| EPI_ISL_464221 | 2020-02-28 |  |
| EPI_ISL_464257 | 2020-03-01 |  |
| EPI_ISL_464270 | 2020-03-02 |  |
| EPI_ISL_464404 | 2020-03-04 |  |
| EPI_ISL_464418 | 2020-03-05 |  |
| EPI_ISL_464565 | 2020-03-06 |  |
| EPI_ISL_464643 | 2020-03-08 |  |
| EPI_ISL_465636 | 2020-05-07 |  |
| EPI_ISL_466310 | 2020-04-08 |  |
| EPI_ISL_466377 | 2020-04-13 |  |
| EPI_ISL_466550 | 2020-04-14 |  |
| EPI_ISL_466907 | 2020-05-04 |  |
| EPI_ISL_466932 | 2020-03-05 |  |
| EPI_ISL_466938 | 2020-03-05 |  |
| EPI_ISL_466993 | 2020-03-12 |  |
| EPI_ISL_467206 | 2020-04-01 |  |
| EPI_ISL_467224 | 2020-04-02 |  |
| EPI_ISL_467248 | 2020-04-02 |  |
| EPI_ISL_468737 | 2020-03-17 |  |
| EPI_ISL_468768 | 2020-03-21 |  |
| EPI_ISL_468784 | 2020-03-08 |  |
| EPI_ISL_468819 | 2020-03-24 |  |
| EPI_ISL_468831 | 2020-03-24 |  |
| EPI_ISL_468836 | 2020-03-17 |  |
| EPI_ISL_468844 | 2020-03-08 |  |
| EPI_ISL_469441 | 2020-05-11 |  |
| EPI_ISL_470172 | 2020-04-21 |  |
| EPI_ISL_472288 | 2020-05-08 |  |
| EPI_ISL_472578 | 2020-03-31 |  |
| EPI_ISL_472807 | 2020-05-15 |  |
| EPI_ISL_473750 | 2020-05-22 |  |
| EPI_ISL_474682 | 2020-05-01 |  |
| EPI_ISL_474903 | 2020-04-03 |  |
| EPI_ISL_475064 | 2020-03-18 |  |
| EPI_ISL_475080 | 2020-03-23 |  |
| EPI_ISL_475306 | 2020-04-23 |  |
| EPI_ISL_475718 | 2020-03-27 |  |
| EPI_ISL_475768 | 2020-03-18 |  |
| EPI_ISL_475890 | 2020-03-28 |  |
| EPI_ISL_475907 | 2020-03-16 |  |
| EPI_ISL_475919 | 2020-03-11 |  |
| EPI_ISL_475933 | 2020-04-02 |  |
| EPI_ISL_476843 | 2020-03-09 |  |
| EPI_ISL_476953 | 2020-04-11 |  |
| EPI_ISL_476962 | 2020-04-11 |  |
| EPI_ISL_476985 | 2020-04-14 |  |
| EPI_ISL_477793 | 2020-05-20 |  |
| EPI_ISL_478110 | 2020-05-27 |  |
| EPI_ISL_481097 | 2020-03-10 |  |
| EPI_ISL_486486 | 2020-04-01 |  |
| EPI_ISL_487600 | 2020-04-22 |  |
| EPI_ISL_487879 | 2020-04-21 |  |
| EPI_ISL_454039 | 2020-03-15 |  |
| EPI_ISL_490227 | 2020-05-30 |  |
| EPI_ISL_490251 | 2020-05-30 |  |
| EPI_ISL_499475 | 2020-03-17 |  |
| EPI_ISL_499753 | 2020-03-15 |  |
| EPI_ISL_499938 | 2020-05-19 |  |
| EPI_ISL_500343 | 2020-04-21 |  |
| EPI_ISL_500347 | 2020-04-21 |  |
| EPI_ISL_507193 | 2020-05-05 |  |
| EPI_ISL_509004 | 2020-03-16 |  |
| EPI_ISL_510108 | 2020-04-03 |  |
| EPI_ISL_510148 | 2020-05-28 |  |
| EPI_ISL_510167 | 2020-03-10 |  |
| EPI_ISL_510339 | 2020-04-20 |  |
| EPI_ISL_510345 | 2020-04-20 |  |
| EPI_ISL_510965 | 2020-03-21 |  |
| EPI_ISL_511105 | 2020-05-05 |  |
| EPI_ISL_511106 | 2020-04-30 |  |
| EPI_ISL_511234 | 2020-03-21 |  |
| EPI_ISL_511253 | 2020-03-24 |  |
| EPI_ISL_511361 | 2020-04-06 |  |
| EPI_ISL_511468 | 2020-03-13 |  |
| EPI_ISL_511491 | 2020-04-08 |  |
| EPI_ISL_511508 | 2020-04-29 |  |
| EPI_ISL_511666 | 2020-03-30 |  |
| EPI_ISL_511726 | 2020-03-25 |  |
| EPI_ISL_511742 | 2020-03-31 |  |
| EPI_ISL_514511 | 2020-04-22 |  |
| EPI_ISL_517092 | 2020-03-26 |  |
| EPI_ISL_528045 | 2020-03-13 |  |
| EPI_ISL_528092 | 2020-03-14 |  |
| EPI_ISL_528103 | 2020-03-15 |  |
| EPI_ISL_528247 | 2020-03-20 |  |
| EPI_ISL_530008 | 2020-04-14 |  |
| EPI_ISL_530091 | 2020-05-21 |  |
| EPI_ISL_532253 | 2020-05-12 |  |
| EPI_ISL_538002 | 2020-04-24 |  |
| EPI_ISL_538714 | 2020-03-25 |  |
| EPI_ISL_539274 | 2020-04-07 |  |
| EPI_ISL_539501 | 2020-03-05 |  |
| EPI_ISL_419237 | 2020-03-07 |  |
| EPI_ISL_539550 | 2020-03-01 |  |
| EPI_ISL_540871 | 2020-05-28 |  |
| EPI_ISL_547458 | 2020-03-08 |  |
| EPI_ISL_418982 | 2020-03-09 |  |
| EPI_ISL_559974 | 2020-04-28 |  |
| EPI_ISL_569316 | 2020-03-24 |  |
| EPI_ISL_569318 | 2020-03-24 |  |
| EPI_ISL_574928 | 2020-03-20 |  |
| EPI_ISL_574946 | 2020-03-16 |  |
| EPI_ISL_574948 | 2020-03-18 |  |
| EPI_ISL_574990 | 2020-03-16 |  |
| EPI_ISL_583609 | 2020-03-15 |  |
| EPI_ISL_583759 | 2020-03-31 |  |
| EPI_ISL_583760 | 2020-03-31 |  |
| EPI_ISL_583763 | 2020-04-05 |  |
| EPI_ISL_583771 | 2020-03-16 |  |
| EPI_ISL_583863 | 2020-04-14 |  |
| EPI_ISL_583866 | 2020-04-14 |  |
| EPI_ISL_591082 | 2020-04-01 |  |
| EPI_ISL_591091 | 2020-04-15 |  |
| EPI_ISL_602492 | 2020-03-22 |  |
| EPI_ISL_602505 | 2020-03-27 |  |
| EPI_ISL_602543 | 2020-03-19 |  |
| EPI_ISL_418243 | 2020-02-28 |  |
| EPI_ISL_451301 | 2020-02-03 |  |

**Supplementary Table 2. Accession IDs and sampling dates of** **Chinese and European sequences from GISAID, included in the dataset.**


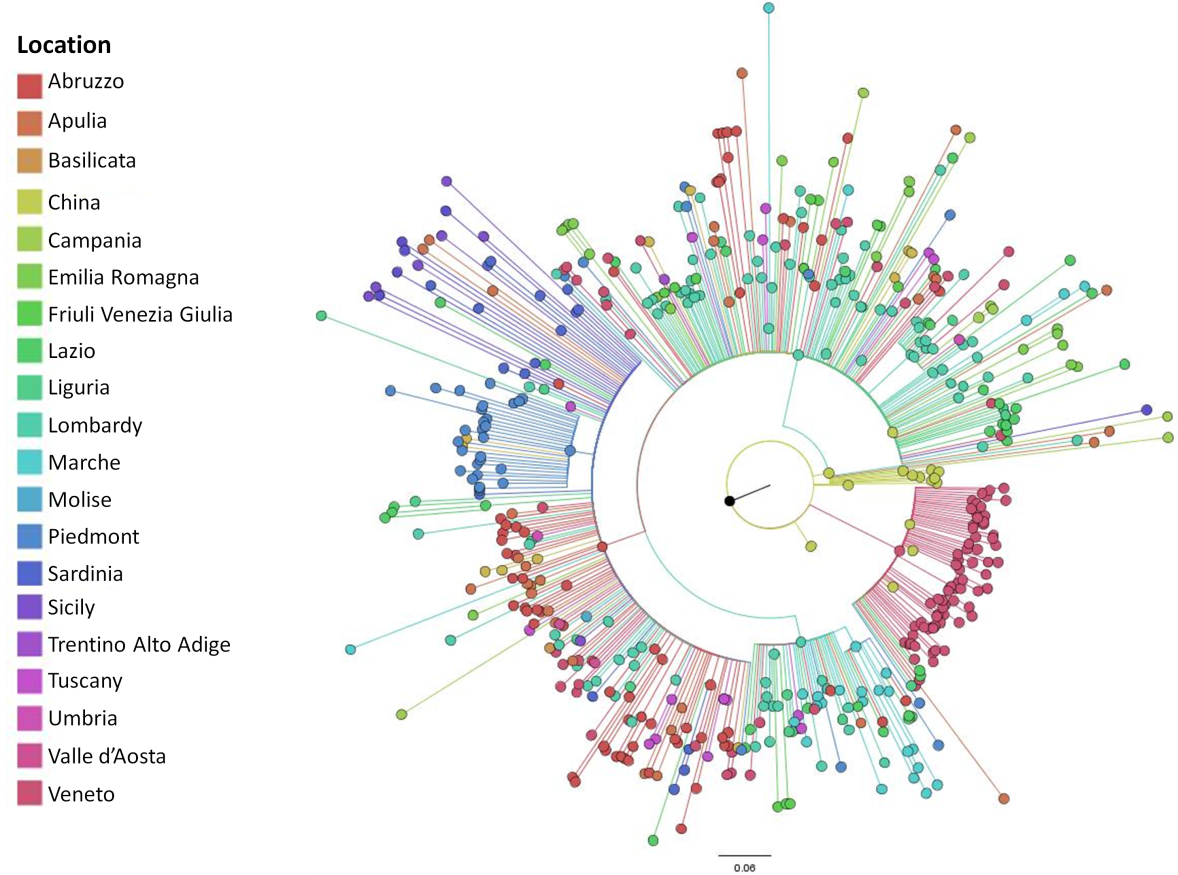


**Supplementary Figure 1. Ancestral reconstruction of SARS-CoV-2 lineages B.1 using the Italian dataset.** The figure show the full tree visualization produced by PastML using marginal posterior probability approximation (MPPA) with an F81-like model.


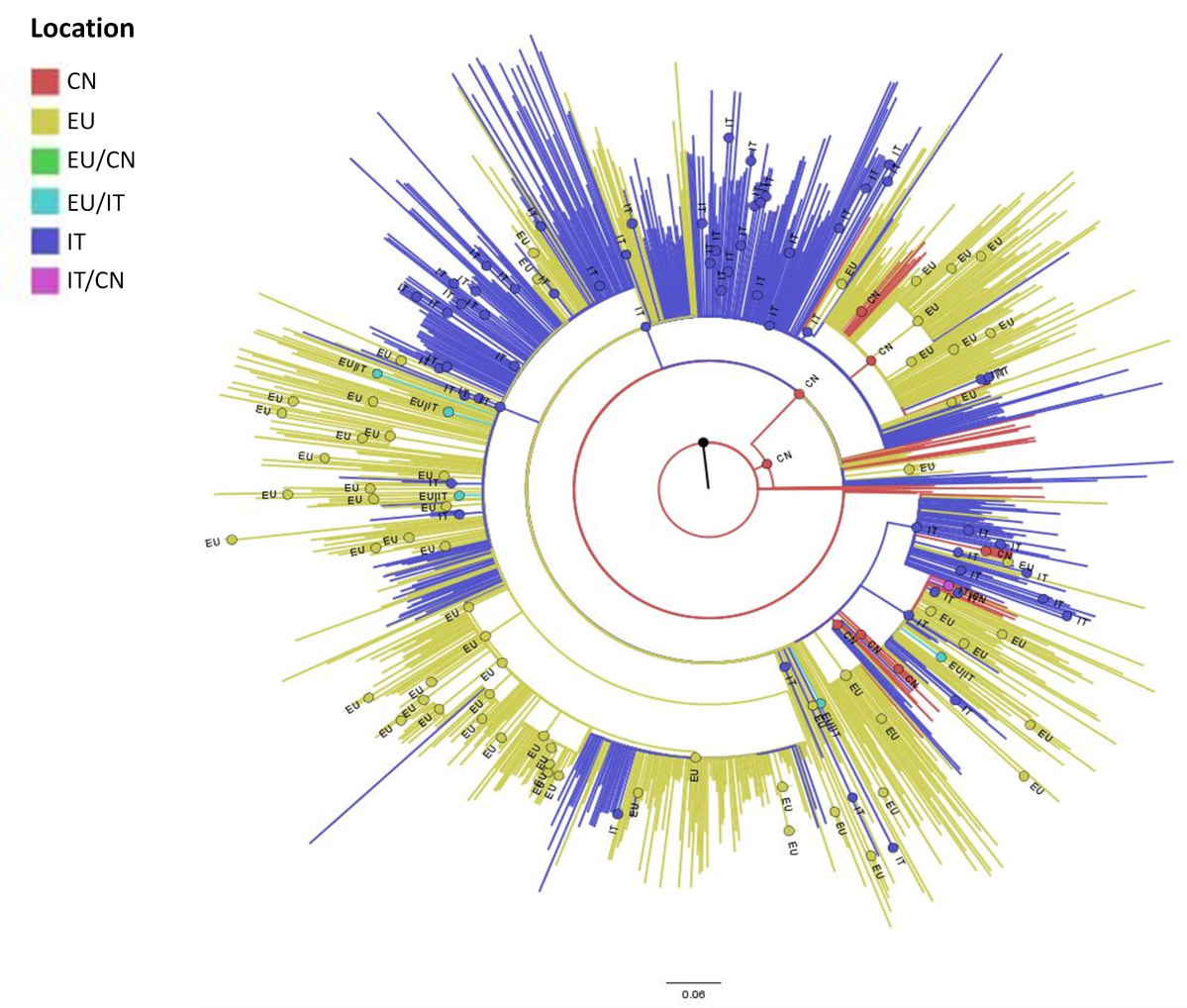


**Supplementary Figure 2. Ancestral reconstruction of SARS-CoV-2 lineages B.1 using the European dataset.** The figure shows the full tree visualization produced by PastML using marginal posterior probability approximation (MPPA) with an F81-like model. CN, China; IT, Italy, EU, Europe.


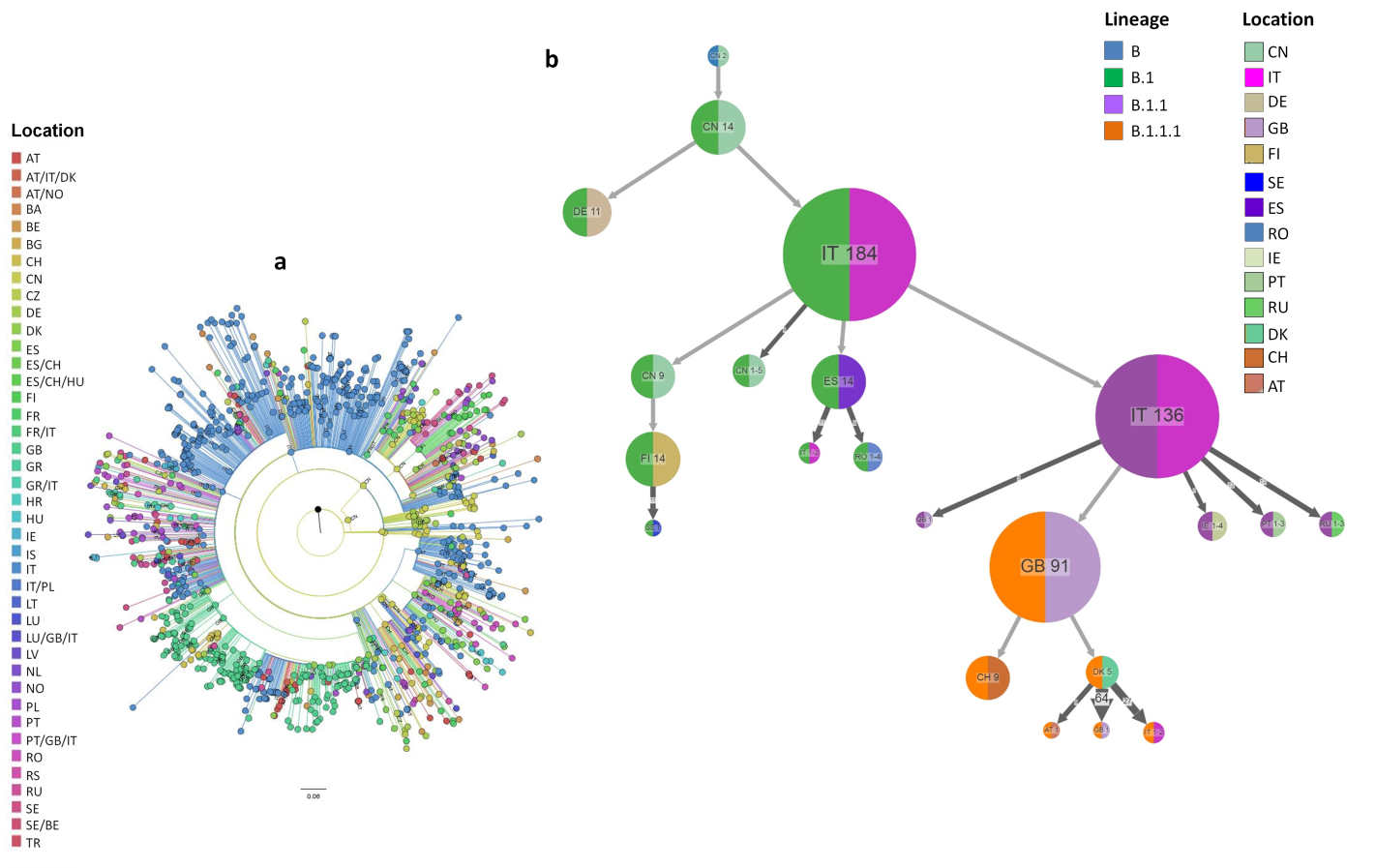


**Supplementary Figure 3. Ancestral reconstruction of SARS-CoV-2 lineages B.1 using the European dataset.** **a**, **b**, The figure shows the full tree (**a**) and compressed visualization produced by PastML (**b**) using marginal posterior probability approximation (MPPA) with an F81-like model. Different colours correspond to different European countries and lineages. Numbers inside (or next to) the circles indicate the number of strains assigned to the specific node. AT, Austria; IT, Italy; DK, Denmark; NO, Norway; BA, Bosnia and Herzegovina; BE, Belgium; BG, Bulgaria; CH, Swiss; CN, China; CZ, Czech Republic; DE, Germany; ES, Spain; HU, Hungary; FI, Finland; FR, France; GB, United Kingdom; GR, Greece; HR, Croatia; IE, Ireland; IS, Iceland; PL, Poland; LT, Lithuania; LU, Luxembourg; LV, Latvia; NL, Netherlands; PT, Portugal; RO, Romania; RS, Serbia; RU, Russia; SE, Sweden; TR, Turkey.


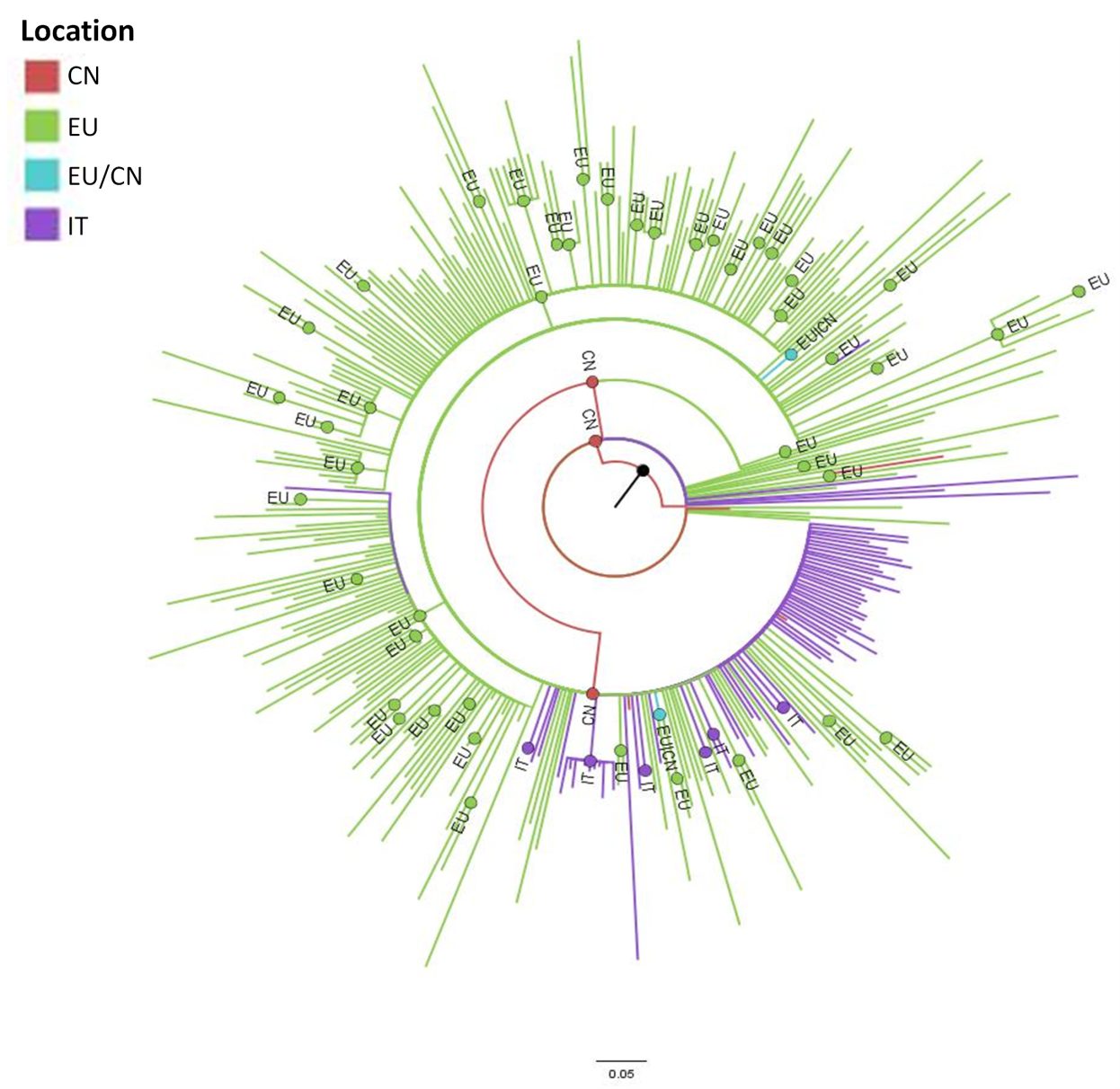


**Supplementary Figure 4. Ancestral reconstruction of SARS-CoV-2 lineages B using the European dataset.** The figure shows the full tree visualization produced by PastML using marginal posterior probability approximation (MPPA) with an F81-like model. CN, China; IT, Italy, EU, Europe.


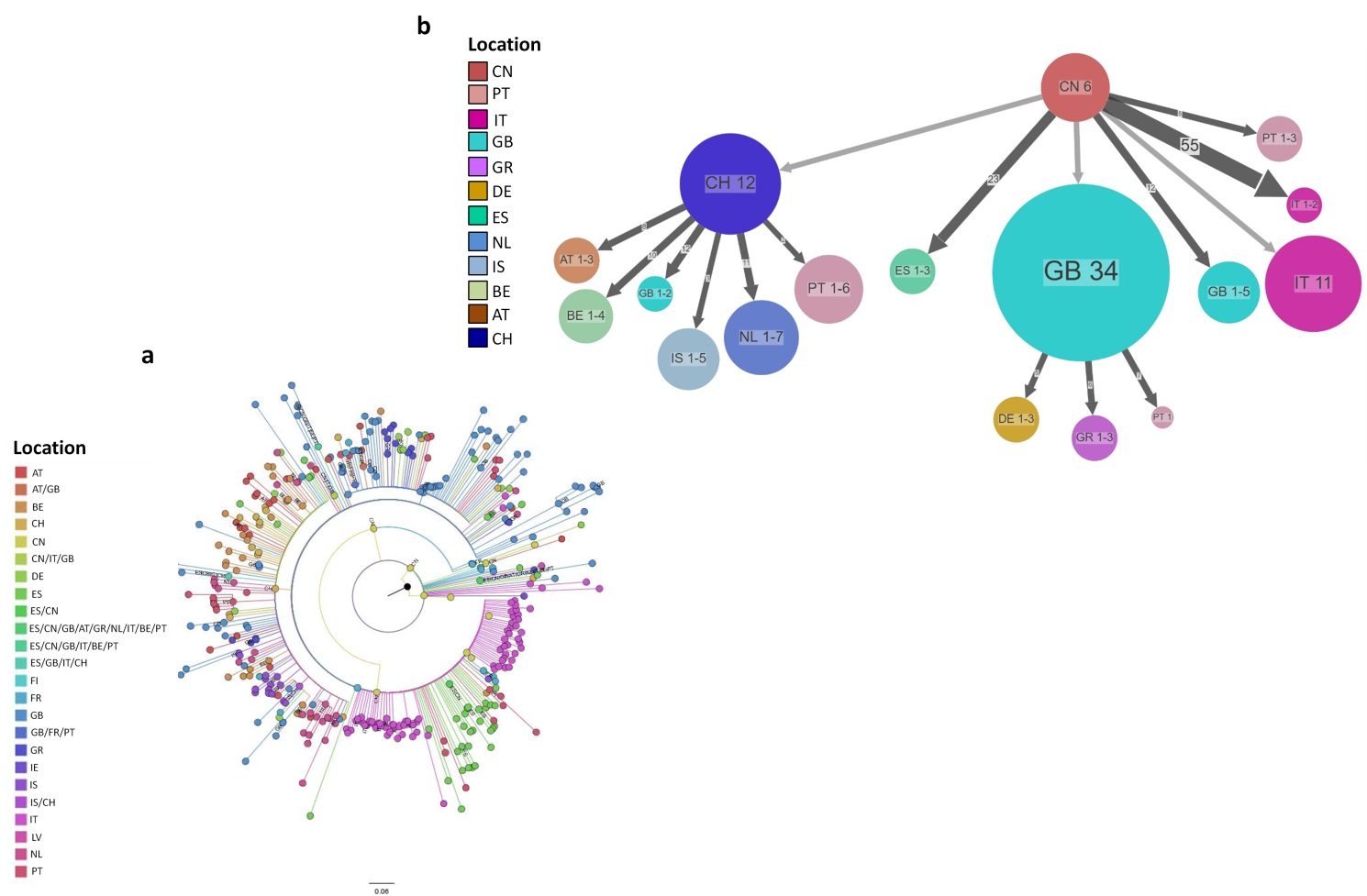


**Supplementary Figure 5.** **Ancestral reconstruction of SARS-CoV-2 lineages B using European dataset.** **a**, **b**, The figure shows the full tree (**a**) and compressed visualization produced by PastML (**b**) using marginal posterior probability approximation (MPPA) with an F81-like model. Different colours correspond to different European countries and lineages. Numbers inside (or next to) the circles indicate the number of strains assigned to the specific node. AT, Austria; GB, United Kingdom; BE, Belgium; CH, Swiss; CN, China; IT, Italy; DE, Germany; ES, Spain; GR, Greece; NL, Netherlands; PT, Portugal; FI, Finland; FR, France; IE, Ireland; IS, Iceland; LV, Latvia.

**Supplementary Figure 6. Genomes distribution in the European and Italian dataset.**
